# Weighted Aronson-Bénilan estimates and Harnack inequalities for slow diffusion equations with a nonlinear forcing term

**DOI:** 10.1007/s00030-026-01190-7

**Published:** 2026-02-16

**Authors:** Ali Taheri, Vahideh Vahidifar

**Affiliations:** School of Mathematical and Physical Sciences, University of Sussex, Falmer, Brighton, United Kingdom

**Keywords:** Smooth metric measure spaces, Nonlinear diffusion, Slow diffusion equation, $\phi$-Laplacian, Aronson-Bénilan estimates, Li-Yau estimates, Harnack inequality, 53C44, 58J60, 58J35, 60J60

## Abstract

We formulate and prove new Aronson-Bénilan and Li-Yau type gradient estimates for positive solutions to nonlinear slow diffusion equations. The framework is that of a smooth metric measure space (i.e., a weighted manifold) and the estimates make use of a range of Harnack quantities with suitable time-variable coefficients. The proofs exploit the intricate relation between geometry, nonlinearity and dynamics of the equation and the results extend, unify and improve various earlier estimates on slow diffusion equations. A number of important corollaries and implications, notably, to parabolic Harnack inequalities and global bounds are presented and discussed.

## Introduction

In this paper we are concerned with gradient estimates and Harnack inequalities for positive solutions to nonlinear diffusion equations of slow diffusion type (or equivalently porous medium type). The gradient estimates of interest are of Aronson-Bénilan and Li-Yau types which are of great importance and application in geometric analysis and diffusion theory. They play a fundamental role in our understanding of the short and long term dynamics of solutions, the interaction between geometry and curvature on the one hand and diffusion on the other, and they are instrumental in the derivation of various qualitative properties of solutions. Some of these include parabolic Harnack inequalities, Liouville results, heat kernel bounds, eigenfunction and spectral bounds and description of ancient/eternal solutions (*see* [3, 12, 21, 23] and [13, 14, 19, 20, 26]).

The framework we study in this paper is that of a smooth metric measure space or a weighted manifold. This is a triple $(\mathscr {M},g,d\mu )$ where $({\mathscr {M}}, g)$ is a complete Riemannian manifold of dimension $n \ge 2$, $d\mu =e^{-\phi } dv_g$ is a positive weighted measure associated with the metric tensor *g* and potential function $\phi$, and $dv_g$ is the Riemannain volume measure on ${\mathscr {M}}$. The diffusion operator associated with the triple $(\mathscr {M},g,d\mu )$ is the $\phi$-Laplacian (that depending on the context is also called the Witten, drifting or weighted Laplacian or even the Nelson diffusion operator). It is defined for $w \in \mathscr {C}^2(\mathscr {M})$ by

1.1 $$\begin{aligned} \Delta _\phi w = e^\phi \textrm{div} (e^{-\phi } \nabla w) = \Delta w - \langle \nabla \phi , \nabla w\rangle , \end{aligned}$$

where $\textrm{div}$, $\nabla$, $\Delta$ are the divergence, gradient and Laplace-Beltrami operators associated with $({\mathscr {M}}, g)$ and $\langle \cdot , \cdot \rangle$ is the Riemannian inner product.

From a stochastic viewpoint, the $\phi$-Laplacian is the infinitesimal generator of the diffusion process $(X_t = X_t^x(\omega ))$ on ${\mathscr {M}}$ arising from the SDE (with *drift*):

1.2 $$\begin{aligned} dX_t = \sqrt{2} dW_t - \nabla \phi (X_t) \, dt. \end{aligned}$$

Here $(W_t=W_t^x(\omega ))$ denotes the Riemannian Brownian motion on ${\mathscr {M}}$ starting at *x*. The backward Kolmogoroff equation for $(X_t = X_t^x(\omega ))$ can then be seen to be the $\phi$-heat equation $\partial _t w = \Delta _\phi w = \Delta w - \langle \nabla \phi , \nabla w \rangle$ while the forward Kolmogoroff equation formulated in terms of the formal adjoint $\Delta _\phi ^\star$ of $\Delta _\phi$ is then the Fokker-Planck equation $\partial _t w = \Delta _\phi ^\star w = \Delta w + \textrm{div} (w\nabla \phi )$.

In this paper we are concerned with nonlinear diffusion equations of porous medium or *slow* diffusion type, associated with $\Delta _\phi$, that for a positive function $u=u(x,t)$, with $x \in {\mathscr {M}}$, $t>0$, can be written in the form

1.3 $$\begin{aligned} \square _p u(x,t) = \partial _t u(x,t) - \Delta _\phi u^p(x,t) = \mathscr {N}(t,x,u(x,t)). \end{aligned}$$

Here $p>1$ is a fixed exponent, and ${\mathscr {N}}={\mathscr {N}}(t,x,u)$ is a sufficiently smooth nonlinearity (or equivalently forcing term) depending only on the space-time variable (*x*, *t*) and the dependent variable $u>0$. We refer the interested reader to [4, 5, 10, 23, 26, 37, 39] and the references therein for more on the porous medium equation.

Our aim here is firstly to formulate and prove parabolic gradient estimates of Aronson-Bénilan and Li-Yau types for positive solutions to (1.3) and secondly to derive some of their implications, notably, Harnack inequalities. Incorporating ${\mathscr {N}}={\mathscr {N}}(t,x,u)$ in (1.3) brings in considerable challenges and as part of our aims we investigate closely how geometry, dynamics and nonlinearity interact with one-another and influence the estimates, and their various consequences.^1^

The most commonly studied nonlinearity for diffusion equations is the logarithmic one ${\mathscr {N}}(t,x,u)={\mathsf A}(t,x) u \log u$, specifically,

1.4 $$\begin{aligned} \square _p u(x,t) = \partial _t u (x,t) - \Delta _\phi u^p (x,t) = {\mathsf A}(t,x) u(x,t) \log u(x,t). \end{aligned}$$

See [2, 5, 11, 38, 39, 41] and the references therein for more. Other classes of equations closely relating to (1.4) include

1.5 $$\begin{aligned} \square _p u(x,t) = \partial _t u (x,t) - \Delta _\phi u^p (x,t) = {\mathsf A}(t,x) u^\alpha (x,t) \Gamma (\log u(x,t)) + {\mathsf B}(t,x) u^\beta (x,t), \end{aligned}$$

with ${\mathscr {N}}(t,x,u) = {\mathsf A}(t,x)u^\alpha \Gamma (\log u) + {\mathsf B}(t,x) u^\beta$. Here $\alpha$, $\beta$ are real exponents, ${\mathsf A}$, ${\mathsf B}$, are space-time coefficients and $\Gamma \in {\mathscr {C}}^2(\mathbb {R}, \mathbb {R})$. Another class of equations are Yamabe type equations that take the form

1.6 $$\begin{aligned} \square _p u(x,t) = \partial _t u (x,t) - \Delta _\phi u^p (x,t) = {\mathsf A}(t,x) u^\alpha (x,t) + {\mathsf B}(t,x) u^\beta (x,t). \end{aligned}$$

A generalisation of (1.6) with a superposition of power-like nonlinearities are equations in the form

1.7 $$\begin{aligned} \square _p u(x,t) = \partial _t u (x,t) - \Delta _\phi u^p(x,t) = \sum _{j=1}^d {\mathsf A}_j(t,x) u^{\alpha _j} (x,t) + \sum _{j=1}^d {\mathsf B}_j(t,x) u^{\beta _j} (x,t), \end{aligned}$$

where by comparison with (1.3) the nonlinearity is given by

1.8 $$\begin{aligned} {\mathscr {N}}(t,x,u) = \sum _{j=1}^d {\mathsf A}_j(t,x) u^{\alpha _j} + \sum _{j=1}^d {\mathsf B}_j(t,x) u^{\beta _j}. \end{aligned}$$

Here ${\mathsf A}_j$, ${\mathsf B}_j$ (with $1 \le j \le d$) are space-time coefficients and $\alpha _j \ge 0$, $\beta _j \le 0$ are real exponents (*see* [29–31, 34]). We refer to [6, 8, 13, 15, 23, 25, 42] and [13, 14, 25, 40] for studies of slow diffusion equations on Riemannian manifolds and smooth metric measure spaces in the absence of a nonlinearity or forcing term and applications and [1, 7, 9, 10, 17, 18, 27, 28, 32] and [33, 35, 36] for other closely related topics.

**Plan of the paper.** The main results are presented in Section 2. Here we formulate the main estimates and Harnack inequalities and we give several examples, remarks and supporting discussion. The remainder of the paper is devoted to the proof of the main results in Section 2. In Section 3 we introduce a Harnack quantity with time-variable coefficients built out of the positive solution to equation (1.3) and describe its evolution. In Section 4 we use the evolution identities and inequalities in Section 3 to prove the local estimate in Theorem 2.1. In Section 5 we give the proof of the Harnack inequalities in Theorem 2.3 and Theorem 2.6. In Section 6 we present the proof of the local estimate in Theorem 2.8. In both Sections 4 and 6 localisation and construction of suitable spatial cut-off functions plays a key role. In Section 7 we give the proof of the Harnack inequalities in Theorem 2.10 and Theorem 2.13. The paper ends with the proof of the global gradient bounds in Theorem 2.15 and Theorem 2.16 in Section 8.

**Notation.** We denote by $d=d(x_1,x_2)$ the Riemannian distance between $x_1$ and $x_2$ on ${\mathscr {M}}$. Fixing an origin or reference point *O* on ${\mathscr {M}}$ we write $\varrho =\varrho (x,O)$ for the geodesic radial variable measuring distance between the variable point *x* and the origin *O*. For $R>0$, $T>0$ fixed we define the space-time cylinder

1.9 $$\begin{aligned} Q_{R,T} (O)\equiv \{ (x, t) | d(x, O) \le R, 0 \le t \le T \} \subset {\mathscr {M}} \times [0, T]. \end{aligned}$$

We denote by $\mathscr {B}_R(O) \subset {\mathscr {M}}$ the geodesic ball of radius $R>0$ centered at *O*. It is evident that in this case we have

1.10 $$\begin{aligned} Q_{R,T} (O) = \mathscr {B}_R(O) \times [0, T] \subset {\mathscr {M}} \times [0, T]. \end{aligned}$$

When the choice of *O* is clear from the context we often abbreviate and write *d*(*x*), $\varrho (x)$ or ${\mathscr {B}}_R$ and $Q_{R,T}$ respectively.

We generally denote derivatives and partial derivatives with subscripts. For instance, for ${\mathscr {G}}={\mathscr {G}}(v)$ or $\Gamma =\Gamma (t,x,v)$ we denote derivatives and partial derivatives with respect to *t*, $x=(x_1, \dots , x_n)$ and *v* by ${\mathscr {G}}_v$, $\Gamma _t$, $\Gamma _x$ or $\Gamma _v$ respectively. For functions of the time variable only, say $\alpha =\alpha (t)$, we denote derivative (in time) with a prime (instead of $\alpha _t$), that is, $\alpha '=d\alpha /dt$. Finally we write $z=z_+ + z_-$ with $z_+=\max (z, 0)$ and $z_-=\min (z, 0)$. Note that $z_+ \ge 0$ and $z_- \le 0$.

**The Ricci tensors** ${\mathscr {R}ic}_\phi ^m(g)$ **and the** $\phi$-**Laplacian** $\Delta _\phi$**.** The Bakry-Èmery *m*-Ricci curvature tensor ${\mathscr {R}ic}^m_\phi (g)$ associated with $({\mathscr {M}}, g, d\mu )$ is defined as

1.11 $$\begin{aligned} {\mathscr {R}ic}^m_\phi (g) := {\mathscr {R}ic}(g) + \nabla \nabla \phi - \frac{\nabla \phi \otimes \nabla \phi }{m-n}, \qquad m \ge n. \end{aligned}$$

Here ${\mathscr {R}ic}(g)$ is the usual Riemannain Ricci curvature tensor of *g*, $\nabla \nabla \phi =\textrm{Hess}(\phi )$ denotes the Hessian of $\phi$, and $m \ge n$ is a constant. For the sake of clarity, in (1.11), when $m=n$, by convention $\phi$ is only allowed to be a constant, thus giving ${\mathscr {R}ic}^n_\phi (g)={\mathscr {R}ic}(g)$. This is the Riemannian context with $\Delta _\phi =\Delta$. We also recall the weighted Bochner-Weitzenböck formula asserting that for any *w* of class ${\mathscr {C}}^3({\mathscr {M}})$ it holds

1.12 $$\begin{aligned} \frac{1}{2} \Delta _\phi |\nabla w|^2 - \langle \nabla w, \nabla \Delta _\phi w \rangle = |\nabla \nabla w|^2 + {\mathscr {R}ic}_\phi (\nabla w, \nabla w), \end{aligned}$$

where ${\mathscr {R}ic}_\phi (g)={\mathscr {R}ic}(g) + \nabla \nabla \phi$. It is not difficult to see that this leads to the *inequality*

1.13 $$\begin{aligned} \frac{1}{2} \Delta _\phi |\nabla w|^2 - \langle \nabla w, \nabla \Delta _\phi w \rangle \ge \frac{1}{m} (\Delta _\phi w)^2 + {\mathscr {R}ic}_\phi ^m (\nabla w, \nabla w), \end{aligned}$$

where the terms $(\Delta _\phi w)^2/m$ and ${\mathscr {R}ic}_\phi ^m(\nabla w, \nabla w)$ replace $|\nabla \nabla w|^2$ and ${\mathscr {R}ic}_\phi (\nabla w, \nabla w)$.

## Statement of the main results

In this section we present the main results of the paper. The proofs will follow in subsequent sections after developing the necessary tools and background. Throughout the paper we set $b = [m(p-1)]/[2+ m(p-1)]$. In view of $p>1$ and $m \ge n \ge 2$ we have $0<b<1$. Note that *m* is a real number (not necessarily an integer). In the course of this paper we shall make use of the transformation $v =p u^{p-1}/(p-1)$ between $u>0$ and $v>0$. As a result we use $\mathscr {G} = \mathscr {G}(t,x,v)$ to denote the rescaled nonlinearity defined by

2.1 $$\begin{aligned} \mathscr {G}(t,x,v)= p [(p-1)v/p]^{\frac{p -2}{p-1}} \mathscr {N} \left( t,x,[(p-1)v/p]^{\frac{1}{p-1}}\right) . \end{aligned}$$

Unless otherwise stated we assume $\alpha = \alpha (t) > 1$, $\beta = \beta (t)$ and $\gamma =\gamma (t) \ge 0$ are functions of class $\mathscr {C}^1[0,T]$ for some $T>0$ fixed. Moreover, $\alpha$ and $\gamma$ are nondecreasing, $\gamma (t)>0$ for $t>0$ and $\gamma (0)=0$.

**The first estimate and Harnack inequality.** Associated with functions $\alpha$, $\beta$, $\gamma$ as described above and for $p>1$, $R>0$, $M\ge 0$ and $K=(p-1)(m-1)k$ we introduce the quantities (for $0<t \le T$)

2.2 $$\begin{aligned}&\mathsf L = \frac{\gamma '(t)}{\gamma (t)} +2M K - \frac{2}{b} \beta (t) +\frac{2 [\alpha (t)-1]}{b \alpha ^2(t)} \beta (t), \end{aligned}$$

2.3 $$\begin{aligned}&\mathsf M = 2 M K \beta (t) -\beta '(t) - \frac{1}{b} \beta ^2(t) - \frac{[\alpha (t)-1]^2}{b\alpha ^2(t)} \beta ^2(t). \end{aligned}$$

Moreover we introduce a set of hypothesis and assumptions on the functions $\alpha$, $\beta$, $\gamma$ that depending on the future relevance and need we shall use. (See, e.g., Remark 2.4.) These are

2.4 $$\begin{aligned}&\frac{2\beta (t) - b\alpha '(t)}{2 [\beta (t)-bMK]} = \alpha (t), \end{aligned}$$

2.5 $$\begin{aligned}&2M K \beta (t) - \beta ' (t) - \frac{1}{b} \beta ^2 (t) =0, \end{aligned}$$

2.6 $$\begin{aligned}&\frac{\gamma '(t)}{\gamma (t)} +2M K - \frac{2}{b} \beta (t) +\frac{2 [\alpha (t)-1]}{b \alpha ^2(t)} \beta (t) \le 0. \end{aligned}$$

For future reference we also formulate the condition that there exists a constant $\lambda >0$ such that for $0<t \le T$,

2.7 $$\begin{aligned} 0 < \frac{\gamma (t)}{2[\alpha (t)-1]}\le \lambda . \end{aligned}$$

With this introduction in place we are now in a position to present the first main result in the paper. The proof will be given in Section 4.

### Theorem 2.1

Let $(\mathscr {M}, g, d\mu )$ be a complete smooth metric measure space with $d\mu =e^{-\phi } dv_g$. Suppose that ${\mathscr {R}ic}_\phi ^m(g) \ge -(m-1) k g$ in $\mathscr {B}_{2R}$ for some $m \ge n$, $k \ge 0$ and $R>0$. Let *u* be a positive solution to equation (1.3) with $p>1$ and set $v =p u^{p-1}/(p-1)$. Then for $\alpha$, $\beta$, $\gamma$ as above satisfying (2.4) and (2.7), any $0<\varepsilon <2[\alpha (t)-1]^2/[b \alpha ^2(t)]$ and every (*x*, *t*) in $Q_{R,T}$ with $t>0$ we have

2.8 $$\begin{aligned}&\left[ \frac{|\nabla v|^2}{v} - \alpha \frac{\partial _t v}{v} + \alpha \frac{\mathscr {G}}{v} -\beta \right] (x,t) \le (b \alpha ^2)^2 \frac{c_1^2 p^2 M \lambda }{R^2 \gamma }\nonumber \\&+ b \alpha ^2 \left\{ \frac{(p-1)M}{R^2} [c_2 +(m-1) c_1(1+R \sqrt{k}) + 2 c_1^2] + \sup _{Q_{2R,T}} [\mathscr {G}_v+\mathsf L] _+ \right\} \nonumber \\&+ \alpha \sqrt{b} \sup _{Q_{2R,T}} \bigg [\frac{b \alpha ^2 (\alpha -1)^2 }{4(\alpha -1)^2- 2\varepsilon b \alpha ^2} \bigg [ \frac{\mathscr {G}}{v} - \mathscr {G}_v - \frac{\alpha (p-1)}{\alpha -1} v\mathscr {G}_{vv} +\frac{(\alpha -1)}{b \alpha ^2} \beta \bigg ]_+^2 \nonumber \\&+\beta \mathscr {G}_v -\alpha (p-1) \Delta _\phi \mathscr {G}^x +\frac{3v^{2/3}}{2\varepsilon ^{1/3}} \left[ (\alpha -1) \frac{|\mathscr {G}_x|}{v}+ \alpha (p-1) |\mathscr {G}_{x v}| \right] ^{4/3}+ \mathsf M\bigg ] _+^{1/2}. \end{aligned}$$

Here $M = \sup v$ over $Q_{2R,T}$, $c_1, c_2 > 0$ are as in (3.27)–(3.28) and $\mathsf L$, $\mathsf M$ and $\lambda$ are as in (2.2)–(2.3) and (2.7).

Subject to the bounds in Theorem 2.1 being global (in space) by passing to the limit $R \nearrow \infty$ we obtain the following global (in space) version of the estimate.

### Theorem 2.2

Let $(\mathscr {M}, g, d\mu )$ be a complete smooth metric measure space with $d\mu =e^{-\phi } dv_g$. If ${\mathscr {R}ic}_\phi ^m(g) \ge -(m-1)kg$ on $\mathscr {M}$ for some $m \ge n$, $k \ge 0$ and *v* is bounded with $M = \sup v$ over $\mathscr {M} \times [0, T]$, then for any $0<\varepsilon <2[\alpha (t)-1]^2/[b \alpha ^2(t)]$ and every $x \in \mathscr {M}$ and $0< t \le T$ we have

2.9 $$\begin{aligned}&\left[ \frac{|\nabla v|^2}{v} - \alpha \frac{\partial _t v}{v} + \alpha \frac{\mathscr {G}}{v} -\beta \right] (x,t) \le b \alpha ^2 \sup _{\mathscr {M} \times [0, T]} [\mathscr {G}_v+\mathsf L] _+ \nonumber \\&+ \alpha \sqrt{b} \sup _{\mathscr {M} \times [0, T]} \bigg [ \frac{b \alpha ^2 (\alpha -1)^2 }{4(\alpha -1)^2- 2\varepsilon b \alpha ^2} \bigg [ \frac{\mathscr {G}}{v} - \mathscr {G}_v - \frac{\alpha (p-1)}{\alpha -1} v\mathscr {G}_{vv} +\frac{(\alpha -1)}{b \alpha ^2} \beta \bigg ]_+^2 \nonumber \\&+\beta \mathscr {G}_v -\alpha (p-1) \Delta _\phi \mathscr {G}^x +\frac{3v^{2/3}}{2\varepsilon ^{1/3}} \left[ (\alpha -1) \frac{|\mathscr {G}_x|}{v}+ \alpha (p-1) |\mathscr {G}_{x v}| \right] ^{4/3}+ \mathsf M\bigg ] _+^{1/2}. \end{aligned}$$

One of the immediate applications of the above estimates is to parabolic Harnack inequalities. Here we present the formulation in the global case. The local version of the inequality is analogous and will be abbreviated. The proof is given in Section 5.

### Theorem 2.3

Under the assumptions of Theorem 2.2, for every $x_1, x_2 \in \mathscr {M}$ and $0<t_1<t_2<T$ we have

2.10 $$\begin{aligned} \frac{v(x_1, t_1)}{v(x_2, t_2)} \le ~ \textrm{exp} \left[ \frac{d^2(x_2, x_1)}{(t_2-t_1)^2} \int _{t_1}^{t_2} \frac{\alpha (t)}{4\underline{M}} \, dt + \int _{t_1}^{t_2} \frac{\beta (t)}{\alpha (t)} \, dt + {\mathsf H} (t_2-t_1) \right] . \end{aligned}$$

Here $\underline{M} = \inf v$ over $\mathscr {M} \times [0, T]$, $d(x_1,x_2)$ is the geodesic distance between $x_1, x_2 \in {\mathscr {M}}$ and $\mathsf H$ is given by

2.11 $$\begin{aligned} \mathsf H=&~ \sup _{\mathscr {M} \times [0, T]} [-\mathscr {G}/v]_+ +b \alpha (T) \sup _{\mathscr {M} \times [0, T]} [\mathscr {G}_v+\mathsf L] _+ \nonumber \\&+ \sqrt{b} \sup _{\mathscr {M} \times [0, T]} \bigg [\frac{b \alpha ^2 (\alpha -1)^2 }{4(\alpha -1)^2- 2\varepsilon b \alpha ^2} \bigg [ \frac{\mathscr {G}}{v} - \mathscr {G}_v - \frac{\alpha (p-1)}{\alpha -1} v\mathscr {G}_{vv} +\frac{(\alpha -1)}{b \alpha ^2} \beta \bigg ]_+^2 \nonumber \\&+\beta \mathscr {G}_v -\alpha (p-1) \Delta _\phi \mathscr {G}^x +\frac{3v^{2/3}}{2\varepsilon ^{1/3}} \left[ (\alpha -1) \frac{|\mathscr {G}_x|}{v}+ \alpha (p-1) |\mathscr {G}_{x v}| \right] ^{4/3}+ \mathsf M\bigg ] _+^{1/2}. \end{aligned}$$

### Remark 2.4

Let us pause briefly to make some useful comments and observations in relation to the above theorems and in particular assumptions (2.4)–(2.7). These will then be used in reaching the conclusions in Theorem 2.5 and Theorem 2.6 formulated below.

- If $\alpha$ is constant then from (2.4) it follows that $\beta$ is constant too. From (2.5) it then follows that either $\beta =0$ (not acceptable as then $\alpha <0$) or $\beta = 2bMK$ in which case again from (2.4) we have $\alpha =2$ (which is acceptable as $\alpha >1$).
- Let us take
  2.12 $$\begin{aligned}&\alpha (t) = 1+\frac{\cosh (MKt) \sinh (MKt)-MKt}{\sinh ^2(MKt)}, \end{aligned}$$
  2.13 $$\begin{aligned}&\beta (t)= b MK [ \coth (MKt) +1]. \end{aligned}$$
  Then $\alpha =\alpha (t)$ is increasing and by an easy inspection $1< \alpha (t) \le 2$ for $t>0$. Moreover $\beta (t) \ge 2 bMK$ for $t \ge 0$ and as for (2.4) we firstly have $2 \beta /b - 2 MK = 2 MK \coth (MKt)\ge 2MK$ and secondly by a direct differentiation that
  2.14 $$\begin{aligned} \frac{2\beta /b- \alpha '}{2 \beta /b -2MK} = \alpha . \end{aligned}$$
  Next regarding (2.5) using $\beta '= b (MK)^2 [1-\coth ^2 (MKt)]$ we have
  2.15 $$\begin{aligned} 2 M K \beta -\beta ' - \frac{1}{b} \beta ^2 =0, \end{aligned}$$
  and so from (2.3) it follows that in this case $\mathsf M=-(\alpha -1)^2\beta ^2/(b\alpha ^2) \le 0$.
- Let $\gamma (t) = \tanh (MKt)$. Then $\gamma (t)>0$ for $t>0$, $\gamma (0)=0$ and $\gamma$ is increasing for $t>0$. Moreover it is easily seen that
  2.16 $$\begin{aligned} \frac{\gamma '}{\gamma } = \frac{MK}{\sinh (MKt) \cosh (MKt)}. \end{aligned}$$
  Finally, referring to (2.6), with the choices $\alpha$, $\beta$ as in (2.12)-(2.13), a straightforward calculation gives
  2.17 $$\begin{aligned} \mathsf L = \frac{\gamma '}{\gamma } - 2 MK \coth (MKt) +\frac{2 (\alpha -1)}{b \alpha ^2} \beta \le 0, \end{aligned}$$
  and referring to (2.7) we have (with $\lambda =3/4$)
  2.18 $$\begin{aligned} 0 < \frac{1}{2} \le \frac{\gamma (t)}{2[\alpha (t)-1]} = \frac{ \tanh (MKt)\sinh ^2(MKt)}{2 [\cosh (MKt) \sinh (MKt)-MKt]} \le \frac{3}{4}. \end{aligned}$$

From Theorem 2.1 and Theorem 2.2 and the remark above regarding the functions $\alpha$, $\beta$ in (2.12)-(2.13) and $\gamma = \tanh (MKt)$ we deduce at once the following result.

### Theorem 2.5

Let $(\mathscr {M}, g, d\mu )$ be a complete smooth metric measure space with $d\mu =e^{-\phi } dv_g$. Suppose that ${\mathscr {R}ic}_\phi ^m(g) \ge -(m-1) k g$ in $\mathscr {B}_{2R}$ for some $m \ge n$, $k \ge 0$ and $R>0$. Let *u* be a positive solution to equation (1.3) with $p>1$ and set $v =p u^{p-1}/(p-1)$. Then for any $0<\varepsilon <2[\alpha (t)-1]^2/[b \alpha ^2(t)]$ and every (*x*, *t*) in $Q_{R,T}$ with $t>0$ we have the local estimate

2.19 $$\begin{aligned}&\left[ \frac{|\nabla v|^2}{v} - \alpha \frac{\partial _t v}{v} + \alpha \frac{\mathscr {G}}{v} -\beta \right] (x,t) \le \frac{12 b^2 c_1^2 p^2 M }{R^2 \tanh (MKt)}\nonumber \\&\quad + 4b \left\{ \frac{(p-1)M}{R^2} [c_2 +(m-1) c_1(1+R \sqrt{k}) + 2 c_1^2] + \sup _{Q_{2R,T}} [\mathscr {G}_v] _+ \right\} \nonumber \\&\quad + 2 \sqrt{b} \sup _{Q_{2R,T}} \bigg [\frac{b \alpha ^2 (\alpha -1)^2 }{4(\alpha -1)^2- 2\varepsilon b \alpha ^2} \bigg [ \frac{\mathscr {G}}{v} - \mathscr {G}_v - \frac{\alpha (p-1)}{\alpha -1} v\mathscr {G}_{vv} +\frac{(\alpha -1)}{b \alpha ^2} \beta \bigg ]_+^2 \nonumber \\&\quad +\beta \mathscr {G}_v -\alpha (p-1) \Delta _\phi \mathscr {G}^x +\frac{3v^{2/3}}{2\varepsilon ^{1/3}} \left[ (\alpha -1) \frac{|\mathscr {G}_x|}{v}+ \alpha (p-1) |\mathscr {G}_{x v}| \right] ^{4/3} - \frac{(\alpha -1)^2}{b\alpha ^2} \beta ^2\bigg ] _+^{1/2}. \end{aligned}$$

Here the functions $\alpha =\alpha (t)$ and $\beta =\beta (t)$ are given by

2.20 $$\begin{aligned}&\alpha (t) = 1+\frac{\cosh (MKt) \sinh (MKt)-MKt}{\sinh ^2(MKt)}, \end{aligned}$$

2.21 $$\begin{aligned}&\beta (t)= b MK [ \coth (MKt) +1]. \end{aligned}$$

Moreover, subject to ${\mathscr {R}ic}_\phi ^m(g) \ge -(m-1)kg$ on $\mathscr {M}$, and *v* being bounded with $M = \sup v$ over $\mathscr {M} \times [0, T]$, for every $x \in \mathscr {M}$ and $0< t \le T$ we have the global estimate

2.22 $$\begin{aligned}&\left[ \frac{|\nabla v|^2}{v} - \alpha \frac{\partial _t v}{v} + \alpha \frac{\mathscr {G}}{v} -\beta \right] (x,t) \le 4 b \sup _{\mathscr {M} \times [0, T]} [\mathscr {G}_v] _+ \nonumber \\&+ 2 \sqrt{b} \sup _{\mathscr {M} \times [0, T]} \bigg [\frac{b \alpha ^2 (\alpha -1)^2 }{4(\alpha -1)^2- 2\varepsilon b \alpha ^2} \bigg [ \frac{\mathscr {G}}{v} - \mathscr {G}_v - \frac{\alpha (p-1)}{\alpha -1} v\mathscr {G}_{vv} +\frac{(\alpha -1)}{b \alpha ^2} \beta \bigg ]_+^2 \nonumber \\&+\beta \mathscr {G}_v -\alpha (p-1) \Delta _\phi \mathscr {G}^x +\frac{3v^{2/3}}{2\varepsilon ^{1/3}} \left[ (\alpha -1) \frac{|\mathscr {G}_x|}{v}+ \alpha (p-1) |\mathscr {G}_{x v}| \right] ^{4/3} - \frac{(\alpha -1)^2}{b\alpha ^2} \beta ^2\bigg ] _+^{1/2}. \end{aligned}$$

The proof of this theorem follows directly from Theorem 2.1 and Theorem 2.2 and the comments made in Remark 2.4. In a similar way we deduce the following Harnack inequality from Theorem 2.3 by using the calculations in Remark 2.4. The proof is presented in Section 5.

### Theorem 2.6

Under the assumptions of Theorem 2.5, for every $x_1, x_2 \in \mathscr {M}$ and $0<t_1<t_2<T$ we have

2.23 $$\begin{aligned} \frac{v(x_1, t_1)}{v(x_2, t_2)} \le&~ \left( \frac{\exp (2MKt_2) -2MKt_2-1}{\exp (2MKt_1) -2MKt_1-1}\right) ^{b/2} \times \nonumber \\&\times \textrm{exp} \left\{ \frac{d^2 (x_1,x_2)}{4 \underline{M} (t_2-t_1)} \left[ 1+\frac{t_2 \coth (MK t_2) -t_1 \coth (MKt_1)}{t_2-t_1}\right] + \mathsf H (t_2 -t_1)\right\} . \end{aligned}$$

Here $\underline{M} = \inf v$ over $\mathscr {M} \times [0, T]$, $d(x_1,x_2)$ is the geodesic distance between $x_1, x_2 \in {\mathscr {M}}$ and $\mathsf H$ is given by

2.24 $$\begin{aligned} \mathsf H=&~ \sup _{\mathscr {M} \times [0, T]} [-\mathscr {G}/v]_+ + 2b \sup _{\mathscr {M} \times [0, T]} [\mathscr {G}_v] _+\nonumber \\&+ \sqrt{b} \sup _{\mathscr {M} \times [0, T]} \bigg [ \frac{b \alpha ^2 (\alpha -1)^2 }{4(\alpha -1)^2- 2\varepsilon b \alpha ^2} \bigg [ \frac{\mathscr {G}}{v} - \mathscr {G}_v - \frac{\alpha (p-1)}{\alpha -1} v\mathscr {G}_{vv} +\frac{(\alpha -1)}{b \alpha ^2} \beta \bigg ]_+^2 \nonumber \\&+\beta \mathscr {G}_v -\alpha (p-1) \Delta _\phi \mathscr {G}^x +\frac{3v^{2/3}}{2\varepsilon ^{1/3}} \left[ (\alpha -1) \frac{|\mathscr {G}_x|}{v}+ \alpha (p-1) |\mathscr {G}_{x v}| \right] ^{4/3}- \frac{(\alpha -1)^2}{b\alpha ^2} \beta ^2\bigg ] _+^{1/2}. \end{aligned}$$

### Remark 2.7

Referring to the local and global estimates in Theorem 2.5 we can observe the following points and special cases.

- If $\mathscr {G} \equiv 0$ the local gradient estimate in (2.19) simplifies considerably and takes the form:
  2.25 $$\begin{aligned} \left[ \frac{|\nabla v|^2}{v} - \alpha \frac{\partial _t v}{v}-\beta \right] (x,t)&\le \frac{12 b^2 c_1 ^2 p^2 M}{R^2\tanh (MKt)} \nonumber \\&+ 4 b \frac{(p-1)M}{R^2} \left[ c_2 +(m-1) c_1(1+R \sqrt{k}) + 2 c_1^2\right] . \end{aligned}$$
  Likewise the global estimate in (2.22) simplifies to the following with the right-hand side being zero:
  2.26 $$\begin{aligned}&\left[ \frac{|\nabla v|^2}{v} - \alpha \frac{\partial _t v}{v}-\beta \right] (x,t) \le 0. \end{aligned}$$
- The same estimates (2.25)–(2.26) hold if only along the solution $v=v(x,t)$ and with $\mathscr {G}=\mathscr {G} (v)$ we have $\mathscr {G}_v \le 0$ and $\mathscr {G}- v\mathscr {G}_v - [\alpha (p-1)/(\alpha -1)]v^2\mathscr {G}_{vv} \le 0$. This follows from certain terms vanishing due to their sings and then letting $\varepsilon \searrow 0$. Clearly as $\mathscr {G}$ is not necessarily zero, the term $\mathscr {G}/v$ will remain on the left-hand side of the estimates.
- The estimates in Theorem 2.5 more generally hold for $\alpha$, $\beta$ and $\gamma$ satisfying the assumptions (2.4)–(2.7) with $\gamma (t)$ replacing $\tanh (MKt)$ on the right-hand side of (2.19).

**The second estimate and Harnack inequality.** Let $\alpha$, $\beta$ and $\gamma$ be as described at the start of the section. For $p>1$, $m \ge 2$, $R>0$, $M\ge 0$ and $K=(p-1)(m-1)k$ we introduce the quantities (for $0<t \le T$)

2.27 $$\begin{aligned}&\tilde{ \mathsf L} = \frac{\gamma '(t)}{\gamma (t)} -\frac{2}{t} - \frac{2 [t\beta (t)- b \alpha (t) (1+MKt)]}{b t \alpha ^2(t)}, \end{aligned}$$

2.28 $$\begin{aligned}&\tilde{\mathsf M} = \frac{b}{t^2} (1+MKt)^2 -\frac{2}{t} \beta (t)-\beta '(t) - \frac{[t \beta (t) -b \alpha (t) (1+MKt)]^2}{b t^2 \alpha ^2(t)}. \end{aligned}$$

For future reference and use we also introduce the following set of assumptions (see, e.g., Remark 2.11):

2.29 $$\begin{aligned}&1+MKt -t\alpha '(t)/2=\alpha (t), \end{aligned}$$

2.30 $$\begin{aligned}&\frac{b}{t^2} (1+MKt)^2 -\frac{2}{t} \beta (t) - \beta '(t) =0, \end{aligned}$$

2.31 $$\begin{aligned}&\frac{\gamma '(t)}{\gamma (t)} -\frac{2}{t} - \frac{2 [t \beta (t) - b \alpha (t) (1+MKt)]}{bt \alpha ^2(t)} \le 0. \end{aligned}$$

With these conditions in place we are now in a position to present the next main result of the paper. The proof will be given in Section 6.

### Theorem 2.8

Let $(\mathscr {M}, g, d\mu )$ be a complete smooth metric measure space with $d\mu =e^{-\phi } dv_g$. Suppose that ${\mathscr {R}ic}_\phi ^m(g) \ge -(m-1) k g$ in $\mathscr {B}_{2R}$ for some $m \ge n$, $k \ge 0$ and $R>0$. Let *u* be a positive solution to equation (1.3) with $p>1$ and set $v =p u^{p-1}/(p-1)$. Then for $\alpha$, $\beta$, $\gamma$ as above satisfying (2.7) and (2.29), any $0<\varepsilon <2[\alpha (t)-1]^2/[b \alpha ^2(t)]$ and every (*x*, *t*) in $Q_{R,T}$ with $t>0$ we have

2.32 $$\begin{aligned}&\left[ \frac{|\nabla v|^2}{v} - \alpha \frac{\partial _t v}{v} + \alpha \frac{\mathscr {G}}{v} -\beta \right] (x,t) \le (b \alpha ^2)^2 \frac{c_1^2 p^2 M \lambda }{R^2 \gamma }\nonumber \\&+ b \alpha ^2 \left\{ \frac{(p-1)M}{R^2} [c_2 +(m-1) c_1(1+R \sqrt{k}) + 2 c_1^2] + \sup _{Q_{2R,T}} [\mathscr {G}_v+\tilde{\mathsf L}] _+ \right\} \nonumber \\&+ \alpha \sqrt{b} \sup _{Q_{2R,T}} \bigg [\frac{b \alpha ^2 (\alpha -1)^2 }{4(\alpha -1)^2- 2\varepsilon b \alpha ^2} \bigg [ \frac{\mathscr {G}}{v} - \mathscr {G}_v - \frac{\alpha (p-1)}{\alpha -1} v\mathscr {G}_{vv} - \frac{2[\beta t-b\alpha (1+MKt)]}{b \alpha ^2 t} \bigg ]_+^2 \nonumber \\&+\beta \mathscr {G}_v- \alpha (p-1) \Delta _ \phi \mathscr {G}^x +\frac{3v^{2/3}}{2\varepsilon ^{1/3}} \left[ (\alpha -1) \frac{|\mathscr {G}_x|}{v} + \alpha (p-1) |\mathscr {G}_{x v}| \right] ^{4/3}+ \tilde{\mathsf M}\bigg ] _+^{1/2}. \end{aligned}$$

Here $M = \sup v$ over $Q_{2R,T}$, $c_1, c_2 > 0$ are as in (3.27)–(3.28) and $\lambda$ and $\tilde{\mathsf L}$, $\tilde{\mathsf M}$ are as in (2.7) and (2.27)–(2.28) respectively.

Subject to the bounds in Theorem 2.8 being global (in space) by passing to the limit $R \nearrow \infty$ we obtain the following global (in space) version of the estimate.

### Theorem 2.9

Let $(\mathscr {M}, g, d\mu )$ be a complete smooth metric measure space with $d\mu =e^{-\phi } dv_g$. If ${\mathscr {R}ic}_\phi ^m(g) \ge -(m-1)kg$ on $\mathscr {M}$ for some $m \ge n$, $k \ge 0$ and *v* is bounded with $M = \sup v$ over $\mathscr {M} \times [0, T]$. Then for any $0<\varepsilon <2[\alpha (t)-1]^2/[b \alpha ^2(t)]$ and every $x \in \mathscr {M}$ and $0< t \le T$ we have

2.33 $$\begin{aligned}&\left[ \frac{|\nabla v|^2}{v} - \alpha \frac{\partial _t v}{v} + \alpha \frac{\mathscr {G}}{v} -\beta \right] (x,t) \le b \alpha ^2 \sup _{\mathscr {M} \times [0, T]} [\mathscr {G}_v+\tilde{\mathsf L}] _+ \nonumber \\&+ \alpha \sqrt{b} \sup _{\mathscr {M} \times [0, T]} \bigg [\frac{b \alpha ^2 (\alpha -1)^2 }{4(\alpha -1)^2- 2\varepsilon b \alpha ^2} \bigg [ \frac{\mathscr {G}}{v} - \mathscr {G}_v - \frac{\alpha (p-1)}{\alpha -1} v\mathscr {G}_{vv} - \frac{2[\beta t-b\alpha (1+MKt)]}{b \alpha ^2 t} \bigg ]_+^2 \nonumber \\&+\beta \mathscr {G}_v - \alpha (p-1) \Delta _ \phi \mathscr {G}^x +\frac{3v^{2/3}}{2\varepsilon ^{1/3}} \left[ (\alpha -1) \frac{|\mathscr {G}_x|}{v} + \alpha (p-1) |\mathscr {G}_{x v}| \right] ^{4/3} + \tilde{\mathsf M}\bigg ] _+^{1/2}. \end{aligned}$$

Again, an immediate applications of the above estimates is to parabolic Harnack inequalities. Here we present the formulation in the global case. The local version of the inequality is analogous and will be abbreviated. The proof is given in Section 7.

### Theorem 2.10

Under the assumptions of Theorem 2.9, for every $x_1, x_2 \in \mathscr {M}$ and $0<t_1<t_2<T$ we have

2.34 $$\begin{aligned} \frac{v(x_1, t_1)}{v(x_2, t_2)} \le ~ \textrm{exp} \left[ \frac{d^2(x_2, x_1)}{(t_2-t_1)^2} \int _{t_1}^{t_2} \frac{\alpha (t)}{4\underline{M}} \, dt + \int _{t_1}^{t_2} \frac{\beta (t)}{\alpha (t)} \, dt + {\mathsf H} (t_2-t_1) \right] . \end{aligned}$$

Here $\underline{M} = \inf v$ over $\mathscr {M} \times [0, T]$, $d(x_1,x_2)$ is the geodesic distance between $x_1, x_2 \in {\mathscr {M}}$ and $\mathsf H$ is given by

2.35 $$\begin{aligned}&\mathsf H = \sup _{\mathscr {M} \times [0, T]} [-\mathscr {G}/v]_+ +b \alpha (T) \sup _{\mathscr {M} \times [0, T]} [\mathscr {G}_v+\tilde{\mathsf L}] _+\nonumber \\&+ \sqrt{b} \sup _{\mathscr {M} \times [0, T]} \bigg [ \frac{b \alpha ^2 (\alpha -1)^2 }{4(\alpha -1)^2- 2\varepsilon b \alpha ^2} \bigg [ \frac{\mathscr {G}}{v} - \mathscr {G}_v - \frac{\alpha (p-1)}{\alpha -1} v\mathscr {G}_{vv} - \frac{2[\beta t-b\alpha (1+MKt)]}{b \alpha ^2 t} \bigg ]_+^2 \nonumber \\&+\beta \mathscr {G}_v - \alpha (p-1) \Delta _ \phi \mathscr {G}^x +\frac{3v^{2/3}}{2\varepsilon ^{1/3}} \left[ (\alpha -1) \frac{|\mathscr {G}_x|}{v} + \alpha (p-1) |\mathscr {G}_{x v}| \right] ^{4/3} + \tilde{\mathsf M} \bigg ] _+^{1/2}. \end{aligned}$$

### Remark 2.11

The following implications and special cases of the estimates in Theorem 2.8 and Theorem 2.9 are worthy of being specifically highlighted:

- There are no constant solutions for $\alpha$ and $\beta$ to equations (2.29) and (2.30).
- Let us take
  2.36 $$\begin{aligned}&\alpha (t) = 1+ \frac{2}{3} MKt, \end{aligned}$$
  2.37 $$\begin{aligned}&\beta (t)= \frac{b}{t} + b MK+ \frac{b}{3} (MK)^2t. \end{aligned}$$
  Then $\alpha =\alpha (t)$ is increasing, $\alpha (t)>1$ for $t>0$ and $\beta (t) \ge bMK(1+2\sqrt{3}/3)$ for $t > 0$. Moreover as for (2.29) and (2.30) a direct calculation gives
  2.38 $$\begin{aligned} 1+MKt -t\alpha '/2=\alpha , \end{aligned}$$
  and
  2.39 $$\begin{aligned} \frac{b}{t^2} (1+MKt)^2 -\frac{2}{t} \beta -\beta ' =0. \end{aligned}$$
  In particular from (2.28) it follows that in this case
  2.40 $$\begin{aligned} \tilde{\mathsf M}=- \frac{[\beta t -b \alpha (1+MKt)]^2}{b\alpha ^2t^2}\le 0. \end{aligned}$$
- Let $\gamma (t) = \tanh (MKt)$. Then $\gamma (t)>0$ for $t>0$, $\gamma (0)=0$ and $\gamma$ is increasing for $t>0$. Moreover, referring to (2.31), with the choices $\alpha$, $\beta$ as in (2.36)-(2.37), a straightforward calculation gives
  2.41 $$\begin{aligned} \tilde{\mathsf L} =\frac{\gamma '}{\gamma } -\frac{2}{t} - \frac{2 [\beta t- b \alpha (1+MKt)]}{b\alpha ^2 t} \le 0, \end{aligned}$$
  and referring to (2.7) we have (with $\lambda =3/4$)
  2.42 $$\begin{aligned} 0 < \frac{\gamma (t)}{2[\alpha (t)-1]} = \frac{ 3\tanh (MKt)}{4 MKt} \le \frac{3}{4}. \end{aligned}$$

From Theorem 2.8 and Theorem 2.9 and the comments above on the functions $\alpha$, $\beta$ in (2.36)-(2.37) and $\gamma = \tanh (MKt)$ we deduce at once the following result.

### Theorem 2.12

Let $(\mathscr {M}, g, d\mu )$ be a complete smooth metric measure space with $d\mu =e^{-\phi } dv_g$. Suppose that ${\mathscr {R}ic}_\phi ^m(g) \ge -(m-1) k g$ in $\mathscr {B}_{2R}$ for some $m \ge n$, $k \ge 0$ and $R>0$. Let *u* be a positive solution to equation (1.3) with $p>1$ and set $v =p u^{p-1}/(p-1)$. Then for any $0<\varepsilon <2[\alpha (t)-1]^2/[b \alpha ^2(t)]$ and every (*x*, *t*) in $Q_{R,T}$ with $t>0$ we have the local estimate

2.43 $$\begin{aligned}&\left[ \frac{|\nabla v|^2}{v} - \alpha \frac{\partial _t v}{v} + \alpha \frac{\mathscr {G}}{v} -\beta \right] (x,t) \le \frac{3c_1^2 (b \alpha ^2)^2 p^2 M }{4R^2 \tanh (MKt)}\nonumber \\&+ b \alpha ^2 \left\{ \frac{(p-1)M}{R^2} [c_2 +(m-1) c_1(1+R \sqrt{k}) + 2 c_1^2] + \sup _{Q_{2R,T}} [\mathscr {G}_v] _+ \right\} \nonumber \\&+ \alpha \sqrt{b} \sup _{Q_{2R,T}} \bigg [\frac{b \alpha ^2 (\alpha -1)^2 }{4(\alpha -1)^2- 2\varepsilon b \alpha ^2} \bigg [ \frac{\mathscr {G}}{v} - \mathscr {G}_v - \frac{\alpha (p-1)}{\alpha -1} v\mathscr {G}_{vv} - \frac{2[\beta t-b\alpha (1+MKt)]}{b \alpha ^2 t} \bigg ]_+^2 \nonumber \\&+\beta \mathscr {G}_v - \alpha (p-1) \Delta _ \phi \mathscr {G}^x +\frac{3v^{\frac{2}{3}}}{2\varepsilon ^{\frac{1}{3}}} \left[ (\alpha -1) \frac{|\mathscr {G}_x|}{v} + \alpha (p-1) |\mathscr {G}_{x v}| \right] ^{\frac{4}{3}}\nonumber \\&- \frac{[\beta t -b \alpha (1+MKt)]^2}{b\alpha ^2t^2}\bigg ] _+^{\frac{1}{2}}. \end{aligned}$$

Here the functions $\alpha =\alpha (t)$ and $\beta =\beta (t)$ are given by

2.44 $$\begin{aligned}&\alpha (t) = 1+ \frac{2}{3} MKt,\end{aligned}$$

2.45 $$\begin{aligned}&\beta (t)= \frac{b}{t} + b MK+ \frac{b}{3} (MK)^2t. \end{aligned}$$

Moreover, subject to ${\mathscr {R}ic}_\phi ^m(g) \ge -(m-1)kg$ on $\mathscr {M}$, and *v* being bounded with $M = \sup v$ over $\mathscr {M} \times [0, T]$, for every $x \in \mathscr {M}$ and $0< t \le T$ we have the global estimate

2.46 $$\begin{aligned}&\left[ \frac{|\nabla v|^2}{v} - \alpha \frac{\partial _t v}{v} + \alpha \frac{\mathscr {G}}{v} -\beta \right] (x,t) \le b \alpha ^2 \sup _{\mathscr {M} \times [0, T]} [\mathscr {G}_v] _+ \nonumber \\&+ \alpha \sqrt{b} \sup _{\mathscr {M} \times [0, T]} \bigg [\frac{b \alpha ^2 (\alpha -1)^2 }{4(\alpha -1)^2- 2\varepsilon b \alpha ^2} \bigg [ \frac{\mathscr {G}}{v} - \mathscr {G}_v - \frac{\alpha (p-1)}{\alpha -1} v\mathscr {G}_{vv} - \frac{2[\beta t-b\alpha (1+MKt)]}{b \alpha ^2 t} \bigg ]_+^2 \nonumber \\&+\beta \mathscr {G}_v - \alpha (p-1) \Delta _ \phi \mathscr {G}^x +\frac{3v^{\frac{2}{3}}}{2\varepsilon ^{\frac{1}{3}}} \left[ (\alpha -1) \frac{|\mathscr {G}_x|}{v} + \alpha (p-1) |\mathscr {G}_{x v}| \right] ^{\frac{4}{3}}\nonumber \\&- \frac{[\beta t -b \alpha (1+MKt)]^2}{b\alpha ^2t^2}\bigg ] _+^{\frac{1}{2}}. \end{aligned}$$

The proof of this theorem follows directly from Theorem 2.8 and Theorem 2.9 and the comments made in Remark 2.11. In much the same way we deduce the following Harnack inequality from Theorem 2.10 by using the calculations in Remark 2.11. The proof is presented in Section 7.

### Theorem 2.13

Under the assumptions of Theorem 2.12, for every $x_1, x_2 \in \mathscr {M}$ and $0<t_1<t_2<T$ we have

2.47 $$\begin{aligned} \frac{v(x_1, t_1)}{v(x_2, t_2)} \le&~ \left[ \frac{t_2}{t_1} \right] ^b \left[ \frac{1 + \frac{2}{3}MK t_2}{1 + \frac{2}{3}MK t_1}\right] ^{-b/4} \times \nonumber \\&\times \textrm{exp} \left\{ \frac{d^2 (x_1,x_2)}{4 \underline{M} (t_2-t_1)} \left[ 1+\frac{1}{3} MK (t_2+t_1)\right] + \left[ \frac{b}{2} MK + \mathsf H \right] (t_2 -t_1)\right\} . \end{aligned}$$

Here $\underline{M} = \inf v$ over $\mathscr {M} \times [0, T]$, $d(x_1,x_2)$ is the geodesic distance between $x_1, x_2 \in {\mathscr {M}}$ and $\mathsf H$ is given by

2.48 $$\begin{aligned}&\mathsf H= \sup _{\mathscr {M} \times [0, T]} [-\mathscr {G}/v]_+ + b \left[ 1+ \frac{2}{3} MKT \right] \sup _{\mathscr {M} \times [0, T]} [\mathscr {G}_v] _+\nonumber \\&+ \sqrt{b} \sup _{\mathscr {M} \times [0, T]} \bigg [ \frac{b \alpha ^2 (\alpha -1)^2 }{4(\alpha -1)^2- 2\varepsilon b \alpha ^2} \bigg [ \frac{\mathscr {G}}{v} - \mathscr {G}_v - \frac{\alpha (p-1)}{\alpha -1} v\mathscr {G}_{vv} - \frac{2[\beta t-b\alpha (1+MKt)]}{b \alpha ^2 t} \bigg ]_+^2 \nonumber \\&+\beta \mathscr {G}_v - \alpha (p-1) \Delta _ \phi \mathscr {G}^x +\frac{3v^{\frac{2}{3}}}{2\varepsilon ^{\frac{1}{3}}} \left[ (\alpha -1) \frac{|\mathscr {G}_x|}{v} + \alpha (p-1) |\mathscr {G}_{x v}| \right] ^{\frac{4}{3}}\nonumber \\&- \frac{[\beta t -b \alpha (1+MKt)]^2}{b\alpha ^2t^2}\bigg ] _+^{\frac{1}{2}}. \end{aligned}$$

### Remark 2.14

Referring to the local and global estimates in Theorem 2.12 we can observe the following points and special cases.

- If $\mathscr {G} \equiv 0$ the local gradient estimate in (2.43) simplifies considerably and takes the form:
  2.49 $$\begin{aligned} \left[ \frac{|\nabla v|^2}{v} - \alpha \frac{\partial _t v}{v}-\beta \right] (x,t)&\le \frac{3 c_1^2 (b \alpha ^2)^2p^2 M }{4R^2 \tanh (MKt)} \nonumber \\&+ b \alpha ^2 \frac{(p-1)M}{R^2} \left[ c_2 +(m-1) c_1(1+R \sqrt{k}) + 2 c_1^2\right] . \end{aligned}$$
  Likewise the global estimate in (2.46) simplifies to the following with the right-hand side being zero:
  2.50 $$\begin{aligned}&\left[ \frac{|\nabla v|^2}{v} - \alpha \frac{\partial _t v}{v}-\beta \right] (x,t) \le 0. \end{aligned}$$
- The same estimates (2.49)–(2.50) hold if only along the solution $v=v(x,t)$ and with $\mathscr {G}= \mathscr {G}(v)$ we have $\mathscr {G}_v \le 0$ and $\mathscr {G}- v\mathscr {G}_v - [\alpha (p-1)/(\alpha -1)]v^2\mathscr {G}_{vv} \le 0$. This follows from certain terms vanishing due to their sings and then letting $\varepsilon \searrow 0$. Clearly as $\mathscr {G}$ is not necessarily zero, the term $\mathscr {G}/v$ will remain on the left-hand side of the estimates.
- The estimates in Theorem 2.12 more generally hold for $\alpha$, $\beta$ and $\gamma$ satisfying the assumptions (2.29)–(2.31) and (2.7) with $\gamma (t)$ replacing $\tanh (MKt)$ on the right-hand side of (2.43).

**Global gradient bounds for** ${\mathscr {M}}$ **closed.** We end this section with two further results on global gradient bounds whose proofs use a different ideas. Note that in both these results ${\mathscr {M}}$ is assumed to be closed.

### Theorem 2.15

Let $(\mathscr {M},g,d\mu )$ be a smooth metric measure space with $\mathscr {M}$ closed and $d\mu =e^{-\phi } dv_g$. Assume ${\mathscr {R}ic}^m_\phi (g) \ge - (m-1) k g$ on ${\mathscr {M}}$ for some $m \ge n$ and $k \ge 0$. Let *u* be a positive solution to (1.3) with $1<p \le 1+1/\sqrt{m-1}$ and set $v =p u^{p-1}/(p-1)$. Assume $\Gamma =\Gamma (v)$ with $v>0$ is a given function of class $\mathscr {C}^2$ and suppose the following conditions hold for some *a*:

- $$\Gamma _v (v) \mathscr {G}(t,x,v) \le 0$$,
- $$\Gamma _v(v) + v \Gamma _{vv}(v) \ge 0$$,
- $$\langle \nabla v, \mathscr {G}_x(t,x,v) \rangle \le 0$$,
- $$2\mathscr {G}_v(t,x,v) + \mathscr {G}(t,x,v)/[(p-1)v] \le a$$.

Then for every $x \in \mathscr {M}$ and $0<t \le T$ we have the gradient bound

2.51 $$\begin{aligned} {[}v^\frac{1}{p-1} |\nabla v|^2] (x,t) \le e^{(2MK+a)t} \left\{ \max _{\mathscr {M}} \left[ v^\frac{1}{p-1}|\nabla v|^2 + \Gamma (v) \right] _{t=0} - \Gamma (v(x,t)) \right\} , \end{aligned}$$

where $$M=\sup v$$ and $$K=(p-1)(m-1) k$$.

Note that when , the conditions in Theorem 2.15 simplify to the following:

- $$\Gamma _v (v) \mathscr {G}(v) \le 0$$,
- $$\Gamma _v(v) + v \Gamma _{vv}(v) \ge 0$$,
- $$2\mathscr {G}_v(v) + \mathscr {G}(v)/[(p-1)v] \le a$$.

### Theorem 2.16

Let $(\mathscr {M},g,d\mu )$ be a smooth metric measure space with $\mathscr {M}$ closed and $d\mu =e^{-\phi } dv_g$. Assume ${\mathscr {R}ic}^m_\phi (g) \ge - (m-1) k g$ on ${\mathscr {M}}$ for some $m \ge n$ and $k \ge 0$. Let *u* be a positive solution to (1.3) with $1<p \le 1+1/\sqrt{m-1}$ and set $v =p u^{p-1}/(p-1)$. Suppose the following conditions hold:

- $$\mathscr {G}(t,x,v) \le 0$$,
- $$\langle \nabla v, \mathscr {G}_x(t,x,v) \rangle \le 0$$,
- $$2\mathscr {G}_v(t,x,v) + \mathscr {G}(t,x,v)/[(p-1)v] \le 0$$.

Then for every $x \in {\mathscr {M}}$ and $0<t \le T$ we have the gradient bound

2.52 $$\begin{aligned} {[}v^\frac{1}{p-1} |\nabla v|^2](x,t) \le \frac{(p-1)(1+2MKt)}{p^2t} \left[ \max _{\mathscr {M}} v^\frac{p}{p-1}(x,0) - v^\frac{p}{p-1}(x,t) \right] , \end{aligned}$$

where $$M=\sup v$$ and $$K=(p-1)(m-1) k$$.

## Evolution under $\mathscr {L} = \partial _t -(p-1) v \Delta _ \phi$ of the Harnack quantity *F*

In this section we derive evolution inequalities for certain nonlinear quantities built from a positive solution $u= u(x,t)$ to equation (1.3). For $p>1$ fixed we consider the evolution operator $\mathscr {L}$ defined by

3.1 $$\begin{aligned} \mathscr {L} = \partial _t -(p-1) v \Delta _ \phi , \end{aligned}$$

where $v(x,t) =p u^{p-1}(x,t)/(p-1)$. Recall that $\mathscr {G}(t,x,v)$ and ${\mathscr {N}}(t,x,u)$ are related by (2.1).

### Lemma 3.1

If *u* is a positive solution to (1.3) then *v* satisfies the equation

3.2 $$\begin{aligned} \mathscr {L} [v] =[\partial _t - (p-1) v \Delta _\phi ] v = |\nabla v|^2+\mathscr {G}(t, x, v). \end{aligned}$$

### Proof

By inverting the relation between *u* and *v*, a straightforward calculation gives

3.3 $$\begin{aligned} \partial _t u = (1/p) [(p-1)v/p]^{\frac{2-p}{p-1}} \partial _t v, \qquad \nabla u^p = [(p-1)v/p]^{\frac{1}{p-1}} \nabla v, \end{aligned}$$

and

3.4 $$\begin{aligned} \Delta _\phi u^p&= (1/p) [(p-1)v/p]^{\frac{2-p}{p-1}}[(p-1) v \Delta v + |\nabla v|^2] - [(p-1)v/p]^{\frac{1}{p-1}}\langle \nabla \phi , \nabla v \rangle \nonumber \\&= (1/p) [(p-1)v/p]^{\frac{2-p}{p-1}}[(p-1) v \Delta _\phi v + |\nabla v|^2]. \end{aligned}$$

Substituting the above in (1.3) and rearranging terms gives the desired conclusion. $\square$

### Lemma 3.2

For a pair of functions *f*, *g* of class $\mathscr {C}^2({\mathscr {M}})$ with *g* nowhere zero we have the identity

3.5 $$\begin{aligned} \mathscr {L} [f/g] = (1/g) \mathscr {L} [f] + 2 (p-1) v \langle \nabla (f/g), \nabla g/g \rangle - (f/g^2) \mathscr {L} [g]. \end{aligned}$$

### Proof

Referring to (3.1) we can write $\mathscr {L} [f/g] = [\partial _t - (p-1) v \Delta _ \phi ] (f/g)$ and so making use of $\Delta _\phi (f/g) =(1/g) \Delta _\phi f - 2 (1/g^2) [\langle \nabla f, \nabla g \rangle - (f/g)|\nabla g|^2] - (f/g^2) \Delta _\phi g$ we have

3.6 $$\begin{aligned} \mathscr {L} [f/g] =&~ [\partial _t - (p-1) v \Delta _ \phi ] (f/g) \nonumber \\ =&~ (1/g) \partial _t f - (f/g^2) \partial _t g - (p-1)v (1/g) \Delta _ \phi f \nonumber \\&+2(p-1) v(1/g^2) [\langle \nabla f, \nabla g \rangle - (f/g) |\nabla g|^2]+ (p-1)v (f/g^2) \Delta _\phi g \nonumber \\ =&~(1/g) \mathscr {L} [f] +2(p-1) v(1/g^2) [\langle \nabla f, \nabla g \rangle \!-\! (f/g) |\nabla g|^2] - (f/g^2) \mathscr {L} [g]. \end{aligned}$$

The conclusion now follows at once. $\square$

### Lemma 3.3

Let *u*, *v*, $\mathscr {G}(t,x,v)$ be as in Lemma 3.1 and consider $F=F(x,t)$ defined by

3.7 $$\begin{aligned} F = \frac{|\nabla v|^2}{v} -\alpha (t) \frac{\partial _t v}{v} + \alpha (t) \frac{\mathscr {G}(t,x,v)}{v} -\beta (t), \end{aligned}$$

where $\alpha =\alpha (t)$, $\beta =\beta (t)$ are arbitrary functions of class $\mathscr {C}^1$. Then *F* satisfies the evolution identity

3.8 $$\begin{aligned} \mathscr {L} [F] =&- [(p-1) \Delta _\phi v]^2 + 2p \langle \nabla F, \nabla v \rangle - 2(p-1) |\nabla \nabla v|^2 -2(p-1) {\mathscr {R}ic}_\phi ^m (\nabla v, \nabla v) \nonumber \\&-2(p-1)\frac{\langle \nabla \phi , \nabla v \rangle ^2}{m-n} -\beta ' (t) -\alpha '(t) \frac{\partial _t v}{v} + [1-\alpha (t)] \left[ \frac{\partial _t v}{v} - \frac{\mathscr {G}(t,x,v)}{v} \right] ^2 \nonumber \\&+\frac{2}{v}[1-\alpha (t)] \langle \nabla \mathscr {G}(t,x,v), \nabla v \rangle - \alpha (t)(p-1) \Delta _\phi \mathscr {G}(t,x,v) \nonumber \\&+[\alpha (t)-1] \frac{|\nabla v|^2}{v} \frac{\mathscr {G}(t,x,v)}{v}+ \alpha '(t) \frac{\mathscr {G}(t,x,v)}{v}. \end{aligned}$$

In particular, setting $b = [m(p-1)]/[2+ m(p-1)]$, *F* satisfies the evolution inequality

3.9 $$\begin{aligned} \mathscr {L} [F] \le&- \frac{1}{b}[(p-1)\Delta _\phi v]^2 + 2p \langle \nabla F, \nabla v \rangle -2(p-1) {\mathscr {R}ic}_\phi ^m (\nabla v, \nabla v)\nonumber \\&-\beta '(t)-\alpha '(t) \frac{\partial _t v}{v} +[1-\alpha (t)] \left[ \frac{\partial _t v}{v} - \frac{\mathscr {G}(t,x,v)}{v} \right] ^2\nonumber \\&+\frac{2}{v}[1-\alpha (t)] \langle \nabla \mathscr {G}(t,x,v), \nabla v\rangle - \alpha (t) (p-1) \Delta _\phi [\mathscr {G}(t,x,v)] \nonumber \\&+[\alpha (t)-1] \frac{|\nabla v|^2}{v} \frac{\mathscr {G}(t,x,v)}{v} + \alpha '(t) \frac{\mathscr {G}(t,x,v)}{v}. \end{aligned}$$

### Proof

Using the linearity of $\mathscr {L}$ and the formulation of *F* in (3.7) we can write

3.10 $$\begin{aligned} \mathscr {L} [F] =&~ \mathscr {L} [|\nabla v|^2/v ] - \alpha (t) \mathscr {L} [\partial _t v/v] + \alpha (t) \mathscr {L} [\mathscr {G}(t,x,v)/v]\nonumber \\&-\alpha '(t) \partial _t v/v + \alpha '(t) \mathscr {G}(t,x,v)/v -\beta ' (t). \end{aligned}$$

We expand the expression on the right-hand side by working out separately the first three terms involving $\mathscr {L}$ via Lemma 3.2. Recalling the weighted Bochner-Weitzenböck formula

3.11 $$\begin{aligned} \Delta _\phi |\nabla v|^2&= 2 |\nabla \nabla v|^2 \!+\! 2 \langle \nabla v ,\nabla \Delta _\phi v \rangle \!+\! 2 {\mathscr {R}ic}_\phi ^m (\nabla v, \nabla v) + 2 \langle \nabla \phi , \nabla v \rangle ^2/(m-n), \end{aligned}$$

we can write

3.12 $$\begin{aligned} \mathscr {L} [|\nabla v|^2] =&~ [\partial _t -(p-1) v \Delta _\phi ] |\nabla v|^2 \nonumber \\ =&~ 2 \langle \nabla v ,\nabla \partial _t v \rangle -2 (p-1) v\times \nonumber \\&\times [ |\nabla \nabla v|^2 + \langle \nabla v ,\nabla \Delta _\phi v \rangle + {\mathscr {R}ic}_\phi ^m(\nabla v, \nabla v) +\langle \nabla \phi , \nabla v \rangle ^2/(m-n)] \nonumber \\ =&~ 2 \left\langle \nabla v, \nabla [(p-1) v \Delta _\phi v + |\nabla v|^2 +\mathscr {G}(t,x,v)] \right\rangle -2 (p-1) v \times \nonumber \\&\times [|\nabla \nabla v|^2 + \langle \nabla v, \nabla \Delta _\phi v \rangle + {\mathscr {R}ic}_\phi ^m (\nabla v, \nabla v) + \langle \nabla \phi , \nabla v \rangle ^2/(m-n)] \nonumber \\ =&~ 2 (p-1) |\nabla v|^2 \Delta _\phi v + 2 (p-1) v \langle \nabla v ,\nabla \Delta _\phi v \rangle + 2\langle \nabla v, \nabla |\nabla v|^2 \rangle \nonumber \\&+2 \langle \nabla v ,\nabla \mathscr {G}(t,x,v) \rangle - 2(p-1) v |\nabla \nabla v|^2 - 2 (p-1) v \langle \nabla v, \nabla \Delta _\phi v \rangle \nonumber \\&-2(p-1)v {\mathscr {R}ic}_\phi ^m (\nabla v, \nabla v) - 2 (p-1) v \langle \nabla \phi , \nabla v \rangle ^2/(m-n)\nonumber \\ =&~ 2 (p-1) |\nabla v|^2 \Delta _\phi v + 2\langle \nabla v, \nabla |\nabla v|^2 \rangle +2 \langle \nabla v, \nabla \mathscr {G}(t,x,v) \rangle \nonumber \\&-2(p-1) v |\nabla \nabla v|^2-2(p-1)v {\mathscr {R}ic}_\phi ^m (\nabla v, \nabla v) \nonumber \\&- 2 (p-1) v \langle \nabla \phi , \nabla v \rangle ^2/(m-n). \end{aligned}$$

Hence, for the first term on the right-hand side of (3.10), upon making note of Lemma 3.2 and substituting from (3.2) and (3.12), we have

3.13 $$\begin{aligned} \mathscr {L} [|\nabla v|^2/v] =&~ (1/v) \mathscr {L} [|\nabla v|^2] +2(p-1)v \langle \nabla (|\nabla v|^2/v ), \nabla v/v \rangle - (|\nabla v|^2/v^2) \mathscr {L} [v] \nonumber \\ =&~2 (p-1) |\nabla v|^2 \Delta _\phi v/v +2 \langle \nabla |\nabla v|^2, \nabla v \rangle /v +2 \langle \nabla v ,\nabla \mathscr {G}(t,x,v) \rangle /v\nonumber \\&-2(p-1) |\nabla \nabla v|^2 -2(p-1) {\mathscr {R}ic}_\phi ^m (\nabla v, \nabla v) \nonumber \\&- 2 (p-1) \langle \nabla \phi , \nabla v \rangle ^2/(m-n) +2(p-1)v \langle \nabla (|\nabla v|^2/v), \nabla v/v \rangle \nonumber \\&- |\nabla v|^4/v^2 - (|\nabla v|^2/v^2) \mathscr {G}(t,x,v). \end{aligned}$$

Similarly, to deal with the second term on the right-hand side of (3.10) we start with

3.14 $$\begin{aligned} \mathscr {L} [\partial _t v] =&~ \partial _t [(p-1) v \Delta _\phi v + |\nabla v|^2 + \mathscr {G}(t,x,v) ]-(p-1) v \Delta _\phi \partial _t v \nonumber \\ =&~ (p-1) \partial _t v \Delta _\phi v +(p-1)v \partial _t \Delta _\phi v \nonumber \\&+ 2 \langle \nabla v ,\nabla \partial _t v \rangle +\partial _t \mathscr {G} (t,x,v) -(p-1) v \Delta _\phi \partial _t v\nonumber \\ =&~(p-1) \partial _t v \Delta _\phi v + 2 \langle \nabla v, \nabla \partial _t v \rangle +\partial _t \mathscr {G} (t,x,v). \end{aligned}$$

Next, by using Lemma 3.2 and substituting from (3.2) and (3.14) we have

3.15 $$\begin{aligned} \mathscr {L} [\partial _t v/v] =&~ (1/v) \mathscr {L} [\partial _t v] +2(p-1)v \langle \nabla (\partial _t v/v), \nabla v/v \rangle - (\partial _t v/v^2) \mathscr {L} [v] \nonumber \\ =&~ (p-1) \partial _t v \Delta _\phi v/v + 2 \langle \nabla v , \nabla \partial _t v\rangle /v + \partial _t \mathscr {G} (t,x,v) /v \nonumber \\&+2(p-1)v \langle \nabla (\partial _t v/v), \nabla v/v \rangle - (\partial _t v/v^2) |\nabla v|^2 - (\partial _t v/v^2) \mathscr {G}(t,x,v). \end{aligned}$$

Finally for the third term on the right-hand side of (3.10), again by using Lemma 3.2 and substituting from (3.2), we can write

3.16 $$\begin{aligned} \mathscr {L} [\mathscr {G}(t,x,v)/v]=&~ (1/v) \mathscr {L} [\mathscr {G}(t,x,v)] -(\mathscr {G}(t,x,v)/v^2) \mathscr {L} [v]\nonumber \\&+2(p-1)v \langle \nabla (\mathscr {G}(t,x,v)/v), \nabla v/v \rangle \nonumber \\ =&~ \partial _t \mathscr {G}(t,x,v)/v -(p-1) \Delta _\phi \mathscr {G}(t,x,v)\nonumber \\&+2(p-1) v \langle \nabla (\mathscr {G}(t,x,v)/v), \nabla v/v \rangle \nonumber \\&- (\mathscr {G}(t,x,v)/v^2) |\nabla v|^2 - [\mathscr {G}(t,x,v)/v]^2. \end{aligned}$$

Substituting (3.13), (3.15) and (3.16) back into (3.10) and rearranging terms gives

3.17 $$\begin{aligned} \mathscr {L} [F] =&~2 (p-1) |\nabla v|^2 \Delta _\phi v /v +2 \langle \nabla |\nabla v|^2, \nabla v \rangle /v + 2 \langle \nabla v ,\nabla \mathscr {G}(t,x,v) \rangle /v \nonumber \\&- 2(p-1) |\nabla \nabla v|^2 -2(p-1) {\mathscr {R}ic}_\phi ^m (\nabla v, \nabla v) - 2 (p-1) \langle \nabla \phi , \nabla v \rangle ^2/(m-n)\nonumber \\&+2(p-1) v \langle \nabla (|\nabla v|^2/v), \nabla v/v \rangle - |\nabla v|^4/v^2 - (|\nabla v|^2/v^2) \mathscr {G}(t,x,v)\nonumber \\&- \alpha (t) (p-1) \partial _t v \Delta _\phi v/v - 2\alpha (t) \langle \nabla v, \nabla \partial _t v \rangle /v -\alpha (t) \partial _t \mathscr {G} (t,x,v)/v\nonumber \\&-2 \alpha (t)(p-1) v \langle \nabla ( \partial _t v/v), \nabla v/v \rangle + \alpha (t) (\partial _t v/v^2) |\nabla v|^2 \nonumber \\&+ \alpha (t) (\partial _t v/v^2) \mathscr {G}(t,x,v) + \alpha (t) \partial _t \mathscr {G}(t,x,v)/v - \alpha (t)(p-1) \Delta _\phi \mathscr {G}(t,x,v) \nonumber \\&+2 \alpha (t)(p-1) v \langle \nabla (\mathscr {G}(t,x,v)/v), \nabla v/v \rangle -\alpha (t) (|\nabla v|^2/v^2) \mathscr {G}(t,x,v)\nonumber \\&- \alpha (t) [\mathscr {G}(t,x,v)/v]^2 -\alpha '(t) \partial _t v/v+ \alpha '(t) \mathscr {G}(t,x,v)/v -\beta ' (t). \end{aligned}$$

Taking into account the cancellation of terms and a further rearrangement of the identity then results in

3.18 $$\begin{aligned} \mathscr {L} [F] =&~ [2 |\nabla v|^2/v - \alpha (t) \partial _t v/v] (p-1) \Delta _\phi v - |\nabla v|^4/v^2 + \alpha (t) \partial _t v |\nabla v|^2/v^2\nonumber \\&-2(p-1) |\nabla \nabla v|^2 + 2 \langle \nabla v ,\nabla \mathscr {G}(t,x,v) \rangle /v -2(p-1) {\mathscr {R}ic}_\phi ^m (\nabla v, \nabla v) \nonumber \\&-2 (p-1) \langle \nabla \phi , \nabla v \rangle ^2/(m-n) -[1+\alpha (t)] |\nabla v|^2 \mathscr {G}(t,x,v)/v^2\nonumber \\&+2(p-1) v\langle \nabla (|\nabla v|^2/v), \nabla v/v \rangle + 2 \langle \nabla v, \nabla |\nabla v|^2 \rangle /v - 2\alpha (t) \langle \nabla \partial _t v, \nabla v \rangle /v\nonumber \\&+ \alpha (t) \partial _t v \mathscr {G}(t,x,v)/v^2 -2 \alpha (t)(p-1) v \langle \nabla (\partial _t v/v), \nabla v/v \rangle \nonumber \\&- \alpha (t)(p-1) \Delta _\phi \mathscr {G}(t,x,v) +2\alpha (t)(p-1)v \langle \nabla (\mathscr {G}(t,x,v)/v), \nabla v/v \rangle \nonumber \\&-\alpha (t) [\mathscr {G}(t,x,v)/v]^2 -\alpha '(t) \partial _t v/v+ \alpha '(t) \mathscr {G}(t,x,v)/v -\beta ' (t). \end{aligned}$$

Recalling that $F = |\nabla v|^2/v -\alpha (t) \partial _t v/v + \alpha (t) \mathscr {G}(t,x,v)/v -\beta (t)$, a close inspection of the terms on the right-hand side, enable us to make use of

3.19 $$\begin{aligned} 2(p-1)v \{&\langle \nabla (|\nabla v|^2/v), \nabla v/v \rangle -\alpha (t) \langle \nabla ( \partial _t v/v), \nabla v/v \rangle \nonumber \\&+\alpha (t) \langle \nabla (\mathscr {G}(t,x,v)/v), \nabla v/v \rangle \} = 2(p-1) \langle \nabla F , \nabla v \rangle , \end{aligned}$$

and

3.20 $$\begin{aligned} 2 \langle \nabla |\nabla v|^2, \nabla v \rangle /v -2 \alpha (t) \langle \nabla \partial _t v, \nabla v \rangle /v =&~ 2 \langle \nabla [(F+ \beta (t))v], \nabla v \rangle /v\nonumber \\&- 2\alpha (t) \langle \nabla v ,\nabla \mathscr {G}(t,x,v) \rangle /v\nonumber \\ =&~ 2 [F+ \beta (t)] |\nabla v|^2/v + 2 \langle \nabla F, \nabla v \rangle \nonumber \\&-2 \alpha (t) \langle \nabla \mathscr {G}(t,x,v), \nabla v\rangle /v. \end{aligned}$$

Putting (3.19) and (3.20) together gives

3.21 $$\begin{aligned}&2(p-1)v \{ \langle \nabla (|\nabla v|^2/v), \nabla v/v \rangle - \alpha (t) \langle \nabla ( \partial _t v/v), \nabla v/v \rangle \nonumber \\&+\alpha (t) \langle \nabla (\mathscr {G}(t,x,v)/v), \nabla v/v \rangle \} + 2\langle \nabla v, \nabla |\nabla v|^2 \rangle /v - 2\alpha (t) \langle \nabla v , \nabla \partial _t v \rangle /v\nonumber \\&= 2p \langle \nabla F, \nabla v \rangle + 2 [F+ \beta (t)] |\nabla v|^2/v - 2\alpha (t) \langle \nabla v ,\nabla \mathscr {G}(t,x,v) \rangle /v\nonumber \\&= 2p \langle \nabla F, \nabla v \rangle +2 \left[ |\nabla v|^2/v -\alpha (t) \partial _t v/v + \alpha (t) \mathscr {G}(t,x,v)/v \right] |\nabla v|^2/v\nonumber \\&-2\alpha (t) \langle \nabla v ,\nabla \mathscr {G}(t,x,v) \rangle /v. \end{aligned}$$

Now by using (3.2), for the first three terms on the right-hand side of (3.18), we can write

3.22 $$\begin{aligned} [ 2 |\nabla v|^2/v - \alpha (t) \partial _t v/v]&(p-1) \Delta _\phi v - |\nabla v|^4/v^2 + \alpha (t)\partial _t v |\nabla v|^2/v^2 \nonumber \\ =&~ [2 |\nabla v|^2/v - \alpha (t) \partial _t v/v] [\partial _t v/v -|\nabla v|^2/v - \mathscr {G}(t,x,v)/v ]\nonumber \\&- |\nabla v|^4/v^2 + \alpha (t) \partial _t v |\nabla v|^2/v^2 \nonumber \\ =&~ 2[\alpha (t)+1] \partial _t v |\nabla v|^2/v^2 - 3 |\nabla v|^4/v^2 - \alpha (t) [\partial _t v/v]^2\nonumber \\&-2 |\nabla v|^2 \mathscr {G}(t,x,v)/v^2 +\alpha (t) \partial _t v \mathscr {G}(t,x,v)/v^2. \end{aligned}$$

Hence, putting (3.21) and (3.22) together, rearranging terms, it follows after some straightforward calculations that

3.23 $$\begin{aligned} 2&(p-1) v \{ \langle \nabla ( |\nabla v|^2/v), \nabla v/v \rangle - \alpha (t) \langle \nabla ( \partial _t v/v), \nabla v/v \rangle \nonumber \\&+\alpha (t) \langle \nabla ( \mathscr {G}(t,x,v)/v), \nabla v/v \rangle \} +2 \langle \nabla v, \nabla |\nabla v|^2 \rangle /v - 2\alpha (t) \langle \nabla v , \nabla \partial _t v \rangle /v\nonumber \\&+ [ 2 |\nabla v|^2/v - \alpha (t) \partial _t v/v ] [ (p-1) \Delta _\phi v ]-|\nabla v|^4/v^2 + \alpha (t) \partial _t v |\nabla v|^2/v^2\nonumber \\ =&~ 2p \langle \nabla F, \nabla v \rangle - [\partial _t v/v-|\nabla v|^2/v -\mathscr {G}(t,x,v)/v ]^2 +[1-\alpha (t)] [\partial _t v/v ]^2 \nonumber \\&+ 2 \alpha (t) |\nabla v|^2 \mathscr {G}(t,x,v)/v^2 + [\mathscr {G}(t,x,v)/v]^2 -2 \alpha (t) \langle \nabla v ,\nabla \mathscr {G}(t,x,v) \rangle /v\nonumber \\&+[\alpha (t)-2] \partial _t v \mathscr {G}(t,x,v)/v^2 \nonumber \\ =&~ 2p \langle \nabla F, \nabla v \rangle - [(p-1) \Delta _\phi v]^2 +[1-\alpha (t)] [\partial _t v/v ]^2+ 2 \alpha (t) |\nabla v|^2 \mathscr {G}(t,x,v)/v^2 \nonumber \\&+ [\mathscr {G}(t,x,v)/v]^2 - 2\alpha (t) \langle \nabla v ,\nabla \mathscr {G}(t,x,v) \rangle /v +[\alpha (t)-2] \partial _t v \mathscr {G}(t,x,v)/v^2. \end{aligned}$$

Now substituting (3.23) back into (3.18) and simplifying terms results in

3.24 $$\begin{aligned} \mathscr {L} [F] =&- 2(p-1) |\nabla \nabla v|^2 -2(p-1) {\mathscr {R}ic}_\phi ^m (\nabla v, \nabla v) -2(p-1)\langle \nabla \phi , \nabla v \rangle ^2/(m-n)\nonumber \\&+ 2p \langle \nabla F, \nabla v \rangle - [(p-1) \Delta _\phi v]^2 +[1-\alpha (t)] [\partial _t v/v]^2\nonumber \\&+2[1-\alpha (t)] \langle \nabla v ,\nabla \mathscr {G}(t,x,v) \rangle /v - \alpha (t)(p-1) \Delta _\phi [\mathscr {G}(t,x,v)]\nonumber \\&+[\alpha (t)-1] |\nabla v|^2\mathscr {G}(t,x,v)/v^2 + 2[\alpha (t)-1] \partial _t v \mathscr {G}(t,x,v)/v^2 \nonumber \\&+[1-\alpha (t)] [\mathscr {G}(t,x,v)/v]^2 + \alpha '(t) \mathscr {G}(t,x,v)/v -\alpha '(t) \partial _t v/v-\beta ' (t). \end{aligned}$$

Finally, making note of the relation

$$\begin{aligned} {[}1-\alpha (t)] \left[ \frac{\partial _t v}{v} \right] ^2 + 2[\alpha (t)-1] \frac{\partial _t v}{v} \frac{\mathscr {G}}{v} +[1-\alpha (t)] \left[ \frac{\mathscr {G}}{v}\right] ^2 =[1-\alpha (t)] \left[ \frac{\partial _t v}{v} - \frac{\mathscr {G}}{v} \right] ^2, \end{aligned}$$

and substituting into (3.24) gives the desired conclusion. The second assertion follows from the first by using the inequality $|\nabla \nabla v|^2+\langle \nabla \phi , \nabla v \rangle ^2/(m-n) \ge (\Delta _\phi v)^2/m$. $\square$

The proof of the gradient estimates in Theorem 2.1 and Theorem 2.8 is based on the use of suitable cut-off functions and localisations. We use the following standard lemma on the existence of a profile function with the relevant properties (*see* [20, 21]).

### Lemma 3.4

There exists a function $\bar{\eta }:[0,\infty ) \rightarrow \mathbb {R}$ satisfying the following properties:

(i) $\bar{\eta }$ is of class $\mathscr {C}^2 [0, \infty )$.
(ii) $0 \le \bar{\eta }(s) \le 1$ for $0 \le s < \infty$ and $\bar{\eta } \equiv 1$ on [0, 1] and $\bar{\eta } \equiv 0$ on $[2, \infty )$.
(iii) $\bar{\eta }' \le 0$ and so $\bar{\eta }$ is non-increasing and for suitable constants $c_1, c_2>0$ we have the global bounds 3.25

Let us now pick a reference point $x_0 \in M$, fix $R, T>0$ and $0<T_1 \le T$. Then, with $\varrho (x)$ denoting the geodesic radial variable with respect to $x_0$, we define

3.26 $$\begin{aligned} \eta (x)=\bar{\eta } (\varrho (x)/R), \qquad x \in {\mathscr {M}}. \end{aligned}$$

Evidently, $0 \le \eta \le 1$ on ${\mathscr {M}}$, $\eta \equiv 1$ on ${\mathscr {B}}_R={\mathscr {B}}_R(x_0)$ and $\eta \equiv 0$ outside ${\mathscr {B}}_{2R}={\mathscr {B}}_{2R}(x_0)$. Moreover, by direct differentiation, using (3.25) and the weighted Laplace comparison theorem (assuming ${\mathscr {R}ic}_\phi (g) \ge -(m-1)k g$ in ${\mathscr {B}}_{2R}$ for some $k \ge 0$, $m \ge n$) we have

3.27 $$\begin{aligned} \frac{|\nabla \eta |^2}{\eta } = \frac{\bar{\eta }'^2}{\bar{\eta }} \frac{|\nabla \varrho |^2}{R^2} \le \frac{c_1 ^2}{R^2}, \end{aligned}$$

3.28 $$\begin{aligned} -\Delta _\phi \eta \le \frac{1}{R^2}[c_2 +(m-1) c_1(1+R \sqrt{k})]. \end{aligned}$$

## Proof of the first local estimate in Theorem 2.1

### Lemma 4.1

Under the assumptions of Lemma 3.3, if ${\mathscr {R}ic}_\phi ^m(g) \ge -(m-1) k g$ and $\alpha (t) \ge 1$, $\beta (t) >bMK$ satisfy $(2\beta - b\alpha ')/(2 \beta -2bMK) = \alpha$, where $M=\sup v$ and $K=(p-1)(m-1) k$, then we have

4.1 $$\begin{aligned} \mathscr {L} [F] \le&- \frac{1}{b\alpha ^2} F^2 - \frac{2(\alpha -1)}{b\alpha ^2} \left[ \frac{|\nabla v|^2}{v} - \beta \right] F - \left[ \frac{2}{b} \beta -2MK - \mathscr {G}_v\right] F \nonumber \\&+ 2p \langle \nabla F, \nabla v \rangle +\left\{ (\alpha -1) \left[ \frac{\mathscr {G}}{v} - \mathscr {G}_v\right] - \alpha (p-1)v \mathscr {G}_{vv} \right\} \frac{|\nabla v|^2}{v}\nonumber \\&- \frac{ (\alpha -1)^2}{b\alpha ^2}\left[ \frac{|\nabla v|^2}{v} - \beta \right] ^2 - \left[ \frac{2}{b} \beta -2MK- \mathscr {G}_v\right] \beta +\frac{1}{b}\beta ^2 -\beta '\nonumber \\&-\alpha (p-1) \Delta _\phi \mathscr {G}^x +\left[ 2 (\alpha -1)\frac{|\mathscr {G}_x|}{v} + 2\alpha (p-1) |\mathscr {G}_{x v}|\right] |\nabla v|. \end{aligned}$$

Here we have abbreviated the arguments of $\mathscr {G}$, $\mathscr {G}^x$ and its partial derivatives $\mathscr {G}_v, \mathscr {G}_{vv}, \mathscr {G}_x, \mathscr {G}_{xv}$ as well as $\alpha , \alpha ', \beta , \beta '$.

### Proof

Starting from (3.9), using the curvature lower bound ${\mathscr {R}ic}_\phi ^m (g) \ge -(m-1)k g$, $0<v\le M$ and setting $K=(p-1)(m-1) k$ we can write

4.2 $$\begin{aligned} \mathscr {L} [F] \le&- \frac{1}{b}[(p-1)\Delta _\phi v+\beta ]^2+\frac{2}{b} \beta (p-1)\Delta _\phi v+\frac{1}{b}\beta ^2 +2MK \frac{|\nabla v|^2}{v}\nonumber \\&+ 2p \langle \nabla F, \nabla v \rangle +\frac{2}{v} (1-\alpha ) \langle \nabla \mathscr {G}, \nabla v \rangle - \alpha (p-1) \Delta _\phi \mathscr {G} \nonumber \\&+(\alpha -1) \frac{|\nabla v|^2}{v} \frac{\mathscr {G}}{v} + \alpha ' \frac{\mathscr {G}}{v} -\alpha ' \frac{\partial _t v}{v} -\beta ' + (1-\alpha ) \left[ \frac{\partial _t v}{v} - \frac{\mathscr {G}}{v} \right] ^2. \end{aligned}$$

Utilising (3.2) and rearranging terms we can write

4.3 $$\begin{aligned} \mathscr {L} [F] \le&- \frac{1}{b}[(p-1)\Delta _\phi v+\beta ]^2+\frac{2}{b} \beta \left[ \frac{\partial _t v}{v}-\frac{|\nabla v|^2}{v}-\frac{\mathscr {G}}{v} \right] +\frac{1}{b}\beta ^2\nonumber \\&+2MK \frac{|\nabla v|^2}{v}+ 2p \langle \nabla F, \nabla v \rangle +\frac{2}{v}(1-\alpha ) \langle \nabla \mathscr {G}, \nabla v \rangle - \alpha (p-1) \Delta _\phi \mathscr {G} \nonumber \\&+(\alpha -1) \frac{|\nabla v|^2}{v} \frac{\mathscr {G}}{v} + \alpha ' \frac{\mathscr {G}}{v} -\alpha ' \frac{\partial _t v}{v} -\beta ' + (1-\alpha ) \left[ \frac{\partial _t v}{v} - \frac{\mathscr {G}}{v} \right] ^2\nonumber \\&= - \frac{1}{b}[(p-1)\Delta _\phi v+\beta ]^2+ 2p \langle \nabla F, \nabla v \rangle +(1-\alpha ) \left[ \frac{\partial _t v}{v} - \frac{\mathscr {G}}{v} \right] ^2\nonumber \\&- \left[ \frac{2}{b} \beta -2MK\right] \left[ \frac{|\nabla v|^2}{v} - \frac{2\beta - b\alpha '}{2 \beta -2bMK} \frac{\partial _t v}{v} + \frac{2\beta - b\alpha '}{2 \beta -2bMK} \frac{\mathscr {G}}{v}-\beta \right] \nonumber \\&+\left\{ (\alpha -1) \left[ \frac{\mathscr {G}}{v} - 2\mathscr {G}_v\right] - \alpha (p-1)v \mathscr {G}_{vv} \right\} \frac{|\nabla v|^2}{v} -\alpha (p-1) \mathscr {G}_v \Delta _\phi v\nonumber \\&- \alpha (p-1) \Delta _\phi \mathscr {G}^x + \frac{2}{v} \langle \nabla v, (1-\alpha ) \mathscr {G}_x -\alpha (p-1) v\mathscr {G}_{xv} \rangle \nonumber \\&- \left[ \frac{2}{b} \beta -2MK\right] \beta +\frac{1}{b}\beta ^2 -\beta ', \end{aligned}$$

where we have used the relations $\nabla \mathscr {G} = \mathscr {G}_x + \mathscr {G}_v \nabla v$ and $\Delta _\phi \mathscr {G} = \Delta _ \phi \mathscr {G}^x + 2\langle \nabla v ,\mathscr {G}_{x v} \rangle + \mathscr {G}_{vv} |\nabla v|^2+ \mathscr {G}_v \Delta _\phi v$. Let us now suppose that the functions $\alpha$ and $\beta$ are such that the following relation holds

4.4 $$\begin{aligned} \frac{2\beta - b\alpha '}{2 \beta -2bMK} = \alpha . \end{aligned}$$

Then substituting back into (4.3) gives

4.5 $$\begin{aligned} \mathscr {L} [F] \le&- \frac{1}{b}[(p-1)\Delta _\phi v+\beta ]^2+ 2p \langle \nabla F, \nabla v \rangle - \left[ \frac{2}{b} \beta -2MK \right] F \nonumber \\&+\left\{ (\alpha -1) \left[ \frac{\mathscr {G}}{v} - 2\mathscr {G}_v\right] - \alpha (p-1)v \mathscr {G}_{vv}\right\} \frac{|\nabla v|^2}{v}-\alpha (p-1) \mathscr {G}_v \Delta _\phi v \nonumber \\&- \alpha (p-1) \Delta _\phi \mathscr {G}^x + \frac{2}{v} \langle \nabla v, (1-\alpha ) \mathscr {G}_x -\alpha (p-1) v\mathscr {G}_{xv} \rangle \nonumber \\&- \left[ \frac{2}{b} \beta -2MK \right] \beta +\frac{1}{b}\beta ^2 -\beta '. \end{aligned}$$

As (3.2) and (3.7) give $(p-1)\Delta _\phi v = [\partial _t v -|\nabla v|^2 - \mathscr {G}]/v =- [F + (\alpha -1)|\nabla v|^2/v + \beta ]/\alpha$, substituting this in (4.5) and rearranging terms leads to

4.6 $$\begin{aligned} \mathscr {L} [F] \le&- \frac{1}{b\alpha ^2} \left[ F + (\alpha -1)\left( \frac{|\nabla v|^2}{v} - \beta \right) \right] ^2 - \left[ \frac{2}{b} \beta -2MK - \mathscr {G}_v\right] F \nonumber \\&+ 2p \langle \nabla F, \nabla v \rangle +\left\{ (\alpha -1) \left[ \frac{\mathscr {G}}{v} - \mathscr {G}_v\right] - \alpha (p-1)v \mathscr {G}_{vv} \right\} \frac{|\nabla v|^2}{v}\nonumber \\&- \alpha (p-1) \Delta _\phi \mathscr {G}^x + \frac{2}{v} \langle \nabla v, (1-\alpha ) \mathscr {G}_x -\alpha (p-1) v\mathscr {G}_{xv} \rangle \nonumber \\&- \left[ \frac{2}{b} \beta -2MK- \mathscr {G}_v\right] \beta +\frac{1}{b}\beta ^2 -\beta '. \end{aligned}$$

Next, referring to the second term in the penultimate line, by using the Cauchy-Schwarz inequality, we have the following upper bound

4.7 $$\begin{aligned} (2/v)\langle \nabla v, (1-\alpha ) \mathscr {G}_x -\alpha (p-1) v \mathscr {G}_{x v} \rangle&\le (2|\nabla v|/|v|)[ |1-\alpha | |\mathscr {G}_x| + \alpha |p-1| |v| | \mathscr {G}_{x v}|] \nonumber \\&\le 2 |\nabla v| [(\alpha -1) |\mathscr {G}_x|/v + \alpha (p-1) |\mathscr {G}_{x v}|] \end{aligned}$$

as $p>1$ and $\alpha \ge 1$. Now making use of the upper bound (4.7) in (4.6) gives at once the desired conclusion. $\square$

Consider $G = \gamma \eta F$ where $\gamma =\gamma (t) \ge 0$ (with $\gamma (t)>0$ for $t>0$ and $\gamma (0)=0$) is to be specified below, $\eta =\eta (x)$ is the cut-off function in (3.26) and *F* is as in (3.7). Let $(x_1,t_1)$ denote the point where *G* attains its maximum over $Q_{2R, T_1}$. We assume $G (x_1, t_1)>0$ as otherwise the estimate immediately follows from $G= \gamma \eta F \le 0$. So in particular $t_1>0$ and from (*ii*) in Lemma 3.4 we have $\varrho (x_1) < 2R$. Therefore at $(x_1, t_1)$ it holds $\partial _t G \ge 0$, $\nabla G =0$, $\Delta _\phi G \le 0$ and so $\mathscr {L} [G] \ge 0$. Substituting from (4.1) gives

4.8 $$\begin{aligned} 0 \le&~ \mathscr {L} [G] = \gamma \eta \mathscr {L} [F] +2 \gamma (p-1) v(|\nabla \eta |^2/\eta ) F - \gamma F (p-1) v \Delta _\phi \eta +\gamma ' \eta F\nonumber \\ \le&- \frac{\gamma \eta }{b\alpha ^2} F^2 - \frac{2(\alpha -1)}{b\alpha ^2} \left[ \frac{|\nabla v|^2}{v} - \beta \right] \gamma \eta F - \left[ \frac{2}{b} \beta -2MK - \mathscr {G}_v\right] \gamma \eta F \nonumber \\&+ 2p \gamma \eta \langle \nabla F, \nabla v \rangle +\left\{ (\alpha -1) \left[ \frac{\mathscr {G}}{v} - \mathscr {G}_v\right] - \alpha (p-1)v \mathscr {G}_{vv} \right\} \gamma \eta \frac{|\nabla v|^2}{v}\nonumber \\&- \frac{ \gamma \eta (\alpha -1)^2}{b\alpha ^2}\left[ \frac{|\nabla v|^2}{v} - \beta \right] ^2 +\left[ 2 (\alpha -1)\frac{|\mathscr {G}_x|}{v} + 2\alpha (p-1) |\mathscr {G}_{x v}|\right] \gamma \eta |\nabla v|\nonumber \\&+\left[ 2MK \beta -\frac{1}{b} \beta ^2 -\beta ' + \mathscr {G}_v \beta -\alpha (p-1) \Delta _\phi \mathscr {G}^x\right] \gamma \eta \nonumber \\&+2 \gamma (p-1) v\frac{|\nabla \eta |^2}{\eta } F -\gamma F (p-1) v \Delta _\phi \eta + \gamma '\eta F. \end{aligned}$$

By making use of $\nabla G= \gamma \nabla (\eta F)=0$ and so $\eta \nabla F= -F \nabla \eta$ at $(x_1, t_1)$ and rewriting the right-hand side in terms of *G* we have

4.9 $$\begin{aligned} 0 \le&- \frac{G^2}{b \alpha ^2 \gamma \eta } - \frac{\gamma \eta (\alpha -1)^2}{b\alpha ^2} \left[ \frac{|\nabla v|^2}{v} - \beta \right] ^2 + 2 p |\nabla v| \frac{|\nabla \eta |}{\eta } G+ \frac{\gamma '}{\gamma }G\nonumber \\&-\frac{2 (\alpha -1)}{b \alpha ^2} \left[ \frac{|\nabla v|^2}{v} - \beta \right] G - \left[ \frac{2}{b} \beta -2MK \right] G +2 (p-1) v\frac{|\nabla \eta |^2}{\eta ^2} G \nonumber \\&- (p-1) v \frac{\Delta _\phi \eta }{\eta } G +\left[ 2MK \beta -\frac{1}{b} \beta ^2 -\beta ' -\alpha (p-1) \Delta _\phi \mathscr {G}^x\right] \gamma \eta \nonumber \\&+ \mathscr {G}_v [ \beta \gamma \eta +G] +\left[ 2 (\alpha -1)\frac{|\mathscr {G}_x|}{v} + 2\alpha (p-1) |\mathscr {G}_{x v}|\right] \gamma \eta |\nabla v| \nonumber \\&+\left\{ (\alpha -1) \left[ \frac{\mathscr {G}}{v} - \mathscr {G}_v\right] - \alpha (p-1)v \mathscr {G}_{vv} \right\} \gamma \eta \frac{|\nabla v|^2}{v}. \end{aligned}$$

Rearranging terms and multiplying through by $\eta$ gives

4.10 $$\begin{aligned} 0 \le&- \frac{G^2}{b \alpha ^2 \gamma } - \frac{ (\alpha -1)^2}{b\alpha ^2} \gamma \eta ^2\frac{|\nabla v|^4}{v^2} -\frac{2 (\alpha -1)}{b \alpha ^2} \frac{|\nabla v|^2}{v} \eta G + 2 p |\nabla v| \frac{|\nabla \eta |}{\sqrt{\eta }} \sqrt{\eta }G\nonumber \\&+\bigg [ \frac{\gamma '}{\gamma } +2MK- \frac{2}{b} \beta +\frac{2 (\alpha -1)}{b \alpha ^2} \beta + \mathscr {G}_v - (p-1) v \frac{\Delta _\phi \eta }{\eta } +2 (p-1) v\frac{|\nabla \eta |^2}{\eta ^2}\bigg ] \eta G\nonumber \\&+\left[ 2MK \beta -\frac{1}{b}\beta ^2 -\beta ' + \mathscr {G}_v \beta -\alpha (p-1) \Delta _\phi \mathscr {G}^x - \frac{(\alpha -1)^2}{b\alpha ^2} \beta ^2\right] \gamma \eta ^2\nonumber \\&+\left[ (\alpha -1) \left( \frac{\mathscr {G}}{v} - \mathscr {G}_v\right) - \alpha (p-1)v \mathscr {G}_{vv} + \frac{(\alpha -1)^2}{b\alpha ^2} \beta \right] \gamma \eta ^2 \frac{|\nabla v|^2}{v}\nonumber \\&+\left[ 2 (\alpha -1)\frac{|\mathscr {G}_x|}{v} + 2\alpha (p-1) |\mathscr {G}_{x v}|\right] \gamma \eta ^2 |\nabla v|. \end{aligned}$$

Let us now proceed by bounding the terms on the right-hand side of (4.10). Towards this end, by making use of the inequality $-\mathsf ax^2 +\mathsf bx \le \mathsf b^2 /(4\mathsf a)$ with *x* being $|\nabla v|/\sqrt{v}$ and $\mathsf a > 0$, and by taking advantage of (3.25) in Lemma 3.4, we can write

4.11 $$\begin{aligned} -\frac{2(\alpha -1)}{b \alpha ^2} \frac{|\nabla v|^2}{v} \eta G + 2p \sqrt{v} \frac{|\nabla \eta |}{\sqrt{\eta }} \frac{|\nabla v|}{\sqrt{v}} \sqrt{\eta }G \le \frac{b p^2 \alpha ^2 v }{2(\alpha -1)} \frac{c_1 ^2}{R^2} G \le \frac{b p^2 \alpha ^2 M}{2(\alpha -1)} \frac{c_1 ^2}{R^2} G. \end{aligned}$$

Next, regarding the last line on the right-hand side of (4.10), an application of Young’s inequality, with $\varepsilon >0$, gives

4.12 $$\begin{aligned} \left[ (\alpha -1) \frac{|\mathscr {G}_x|}{v}+ \alpha (p-1) |\mathscr {G}_{x v}| \right] |\nabla v|&\le \frac{3v^{2/3}}{4\varepsilon ^{1/3}} \left[ (\alpha -1) \frac{|\mathscr {G}_x|}{v}+ \alpha (p-1) |\mathscr {G}_{x v}| \right] ^{4/3}\nonumber \\&\quad + \varepsilon \frac{|\nabla v|^4}{4v^2}. \end{aligned}$$

Now substituting (4.11)–(4.12) back into (4.10) and rearranging terms results in

4.13 $$\begin{aligned} 0 \le&- \frac{G^2}{b \alpha ^2 \gamma } - \left[ \frac{ (\alpha -1)^2}{b\alpha ^2}- \frac{\varepsilon }{2} \right] \gamma \eta ^2\frac{|\nabla v|^4}{v^2} + \frac{b p^2 \alpha ^2 M}{2(\alpha -1)} \frac{c_1 ^2}{R^2} G\nonumber \\&+\bigg [ \frac{\gamma '}{\gamma } +2MK- \frac{2}{b} \beta +\frac{2 (\alpha -1)}{b \alpha ^2} \beta + \mathscr {G}_v - (p-1) v \frac{\Delta _\phi \eta }{\eta } +2 (p-1) v\frac{|\nabla \eta |^2}{\eta ^2}\bigg ] \eta G\nonumber \\&+\left[ 2MK \beta -\frac{1}{b}\beta ^2 -\beta ' + \mathscr {G}_v \beta -\alpha (p-1) \Delta _\phi \mathscr {G}^x - \frac{(\alpha -1)^2}{b\alpha ^2} \beta ^2\right] \gamma \eta ^2\nonumber \\&+\left[ (\alpha -1) \left( \frac{\mathscr {G}}{v} - \mathscr {G}_v\right) - \alpha (p-1)v \mathscr {G}_{vv} + \frac{(\alpha -1)^2}{b\alpha ^2} \beta \right] \gamma \eta ^2 \frac{|\nabla v|^2}{v}\nonumber \\&+\frac{3v^{2/3}}{2\varepsilon ^{1/3}} \left[ (\alpha -1) \frac{|\mathscr {G}_x|}{v} + \alpha (p-1) |\mathscr {G}_{x v}| \right] ^{4/3}\gamma \eta ^2. \end{aligned}$$

Using the inequality $-\mathsf ax^2 +\mathsf bx \le \mathsf b^2 /(4\mathsf a)$ with *x* being $|\nabla v|^2/v$ and $\mathsf a > 0$ (and with the improvement $- \mathsf a x^2 + \mathsf b x \le 0$ for $\mathsf b \le 0$) and noting $0<\varepsilon <2(\alpha -1)^2/(b \alpha ^2)$, we have

4.14 $$\begin{aligned} -&\frac{2(\alpha -1)^2- \varepsilon b \alpha ^2}{2b \alpha ^2} \frac{|\nabla v|^4}{v^2} + \bigg [(\alpha -1)\left( \frac{\mathscr {G}}{v} -\mathscr {G}_v \right) - \alpha (p-1) v\mathscr {G}_{vv} +\frac{(\alpha -1)^2}{b \alpha ^2} \beta \bigg ] \frac{|\nabla v|^2}{v} \nonumber \\ \le&~ \frac{b \alpha ^2 (\alpha -1)^2 }{4(\alpha -1)^2- 2\varepsilon b \alpha ^2} \bigg [\frac{(\alpha -1)[\mathscr {G} -v\mathscr {G}_v]-\alpha (p-1)v^2\mathscr {G}_{vv}}{(\alpha -1)v} +\frac{(\alpha -1)}{b \alpha ^2} \beta \bigg ]_+^2. \end{aligned}$$

Using the bounds (3.27)–(3.28) on the cut-off function $\eta$ and $0<v\le M$ along with (4.14) from above, substituting these in (4.13) and lastly multiplying through by $\gamma$ it follows that

4.15 $$\begin{aligned} 0 \le&- \frac{G^2}{b \alpha ^2} + \bigg \{\frac{b \alpha ^2 p^2 M \gamma }{2(\alpha -1)} \frac{c_1 ^2}{R^2} +\frac{(p-1)M \gamma }{R^2} [c_2 +(m-1) c_1(1+R \sqrt{k})+ 2 c_1^2] \nonumber \\&+ \gamma \eta \left[ \frac{\gamma '}{\gamma }+2MK - \frac{2}{b} \beta +\frac{2 (\alpha -1)}{b \alpha ^2} \beta + \mathscr {G}_v\right] _+ \bigg \} G +\bigg \{2MK \beta -\frac{1}{b}\beta ^2 -\beta ' +\beta \mathscr {G}_v \nonumber \\&-\alpha (p-1) \Delta _\phi \mathscr {G}^x - \frac{(\alpha -1)^2}{b\alpha ^2} \beta ^2 + \frac{3v^{2/3}}{2\varepsilon ^{1/3}} \left[ (\alpha -1) \frac{|\mathscr {G}_x|}{v}+ \alpha (p-1) |\mathscr {G}_{x v}| \right] ^{4/3}\nonumber \\&+\frac{b \alpha ^2 (\alpha -1)^2 }{4(\alpha -1)^2- 2\varepsilon b \alpha ^2} \bigg [\frac{(\alpha -1)[\mathscr {G} -v\mathscr {G}_v]-\alpha (p-1)v^2\mathscr {G}_{vv}}{(\alpha -1)v} +\frac{(\alpha -1)}{b \alpha ^2} \beta \bigg ]_+^2\bigg \}\gamma ^2 \eta ^2. \end{aligned}$$

Let us denote by $\textbf{I}$ and $\textbf{II}$ the coefficients of *G* and $\gamma ^2 \eta ^2=\gamma ^2 \eta ^2 G^0$ on the right-hand side of (4.15). Thus

4.16 $$\begin{aligned} \textbf{I} =&~ \frac{b \alpha ^2 p^2 M \gamma }{2(\alpha -1)} \frac{c_1 ^2}{R^2} +\frac{(p-1)M \gamma }{R^2} [c_2 +(m-1) c_1(1+R \sqrt{k})+ 2 c_1^2]\nonumber \\&+ \gamma \eta \left[ \frac{\gamma '}{\gamma } +2M K - \frac{2}{b} \beta +\frac{2 (\alpha -1)}{b \alpha ^2} \beta + \mathscr {G}_v \right] _+, \end{aligned}$$

4.17 $$\begin{aligned} \textbf{II} =&~2M K \beta -\beta ' -\frac{1}{b}\beta ^2 - \frac{(\alpha -1)^2}{b\alpha ^2} \beta ^2 + \beta \mathscr {G}_v \nonumber \\&-\alpha (p-1) \Delta _\phi \mathscr {G}^x + \frac{3v^{2/3}}{2\varepsilon ^{1/3}} \left[ (\alpha -1) \frac{|\mathscr {G}_x|}{v}+ \alpha (p-1) |\mathscr {G}_{x v}| \right] ^{4/3}\nonumber \\&+\frac{b \alpha ^2 (\alpha -1)^2 }{4(\alpha -1)^2- 2\varepsilon b \alpha ^2} \bigg [\frac{(\alpha -1)[\mathscr {G} -v\mathscr {G}_v]-\alpha (p-1)v^2\mathscr {G}_{vv}}{(\alpha -1)v} +\frac{(\alpha -1)}{b \alpha ^2} \beta \bigg ]_+^2, \end{aligned}$$

and so (4.15) can be re-written as $0 \le - G^2/(b \alpha ^2) + \textbf{I} G+ \textbf{II} \gamma ^2 \eta ^2$. With the aid of the constants and quantities described above let us introduce the convenient notation

4.18 $$\begin{aligned}&\mathsf L = \frac{\gamma '}{\gamma } +2M K - \frac{2}{b} \beta +\frac{2 (\alpha -1)}{b \alpha ^2} \beta , \end{aligned}$$

4.19 $$\begin{aligned}&\mathsf M = 2 M K \beta -\beta ' - \frac{1}{b} \beta ^2 - \frac{(\alpha -1)^2}{b\alpha ^2} \beta ^2, \end{aligned}$$

and subsequently

4.20 $$\begin{aligned} \mathsf A = \overbrace{b \alpha ^2 p^2 M \lambda \frac{c_1 ^2}{R^2}}^{{\mathsf C}={\mathsf C}(t)} + \underbrace{\gamma \left\{ \frac{(p-1)M}{R^2} [c_2 +(m-1) c_1(1+R \sqrt{k}) + 2 c_1^2] + \sup _{Q_{2R,T}} [\mathscr {G}_v+\mathsf L] _+ \right\} }_{\gamma (t) {\mathsf D}} \end{aligned}$$

and

4.21 $$\begin{aligned} \mathsf B =&~ \sup _{Q_{2R,T}} \bigg [\frac{b \alpha ^2 (\alpha -1)^2 }{4(\alpha -1)^2- 2\varepsilon b \alpha ^2} \left[ \frac{(\alpha -1)[\mathscr {G} -v\mathscr {G}_v]-\alpha (p-1)v^2\mathscr {G}_{vv}}{(\alpha -1)v} +\frac{(\alpha -1)}{b \alpha ^2} \beta \right] _+^2 \nonumber \\&+\beta \mathscr {G}_v-\alpha (p-1) \Delta _\phi \mathscr {G}^x + \frac{3v^{2/3}}{2\varepsilon ^{1/3}} \left[ (\alpha -1) \frac{|\mathscr {G}_x|}{v}+ \alpha (p-1) |\mathscr {G}_{x v}| \right] ^{4/3} +\mathsf M \bigg ]_+. \end{aligned}$$

Then in view of (4.16)–(4.17) and upon recalling (2.7) we have $\textbf{I} \le \mathsf A(t_1)$ and $\textbf{II} \le \mathsf B$ and thus (4.15) gives,

4.22 $$\begin{aligned} 0 \le -\frac{1}{b \alpha ^2} G^2 + \mathsf A (t_1) G + \gamma ^2 \mathsf B. \end{aligned}$$

We now make use of the implication $-\mathsf a x^2 +\mathsf bx +\mathsf c \ge 0 \implies x \le \mathsf b/\mathsf a +\sqrt{\mathsf c/\mathsf a}$ (with ${\mathsf a}>0$, ${\mathsf b}, {\mathsf c} \ge 0$) to deduce that

4.23 $$\begin{aligned} G(x_1, t_1)&\le b \alpha ^2(t_1) \mathsf A (t_1) + \alpha (t_1) \gamma (t_1) \sqrt{b \mathsf B}\nonumber \\&= b \alpha ^2(t_1)[\mathsf C (t_1) + \gamma (t_1) \mathsf D]+ \alpha (t_1) \gamma (t_1) \sqrt{b \mathsf B}. \end{aligned}$$

Since $\eta \equiv 1$ for $d(x,x_0, T_1) \le R$ and $(x_1, t_1)$ is the point where $G= \gamma \eta F$ attains its maximum on $Q_{2R, T_1}$ we have

4.24 $$\begin{aligned} G(x, T_1)= \gamma (T_1) F(x, T_1) = [\gamma \eta F](x, T_1) \le [\gamma \eta F](x_1, t_1) = G(x_1, t_1). \end{aligned}$$

Hence making note of the bound (4.23) and dividing through by $T_1 >0$ gives

4.25 $$\begin{aligned} F(x,T_1) \le \frac{1}{\gamma (T_1)} G(x_1, t_1)&\le b \alpha ^2(t_1)\left[ \frac{\mathsf C (t_1)}{\gamma (T_1)} + \frac{\gamma (t_1)}{\gamma (T_1)} \mathsf D\right] + \alpha (t_1) \frac{\gamma (t_1)}{\gamma (T_1)} \sqrt{b \mathsf B}, \end{aligned}$$

and so in view of $1< \alpha (t_1)\le \alpha (T_1)$ and $0< \gamma (t_1) \le \gamma (T_1)$ due to the monotonicity of $\alpha$ and $\gamma$ this gives

4.26 $$\begin{aligned} F(x,T_1)&\le b \alpha ^2(t_1) \frac{\mathsf C(t_1)}{\gamma (T_1)} +\frac{\gamma (t_1)}{\gamma (T_1)} [b \alpha ^2(t_1) \mathsf D + \alpha (t_1) \sqrt{b \mathsf B}] \nonumber \\&\le b \alpha ^2(T_1) \frac{\mathsf C(T_1)}{\gamma (T_1)} +b \alpha ^2(T_1) \mathsf D + \alpha (T_1) \sqrt{b \mathsf B}. \end{aligned}$$

The arbitrariness of $T_1 >0$ gives the desired conclusion for any $0< t \le T$. $\square$

## Proof of the Harnack inequalities in Theorem 2.3 and Theorem 2.6

In this section we present the proof of the Harnack inequalities resulting from the first gradient estimates in Theorem 2.1 and Theorem 2.5.

### Proof of Theorem 2.3

As this is a global Harnack inequality the idea is to integrate the global estimate (2.9) along suitable space-time curves in $\mathscr {M} \times [0, T]$. To this end, let us more generally proceed by re-writing the latter inequality as

5.1 $$\begin{aligned} \frac{|\nabla v|^2}{v} - \alpha (t) \frac{\partial _t v}{v} - \mathsf h (t) \le 0, \end{aligned}$$

or

5.2 $$\begin{aligned} - \frac{\partial _t v}{v} \le \frac{1}{\alpha (t)} \left( \mathsf h (t) -\frac{|\nabla v|^2}{v}\right) , \end{aligned}$$

where the choice of $\mathsf h(t)$ will be specified below. Now upon setting $f= \log v$ and $\underline{M} = \inf v$ where the infimum is taken over $\mathscr {M} \times [0, T]$ we have

5.3 $$\begin{aligned} - \partial _t f = - \frac{\partial _t v}{v}&\le \frac{1}{\alpha (t)} \left( \mathsf h (t) -\frac{|\nabla v|^2}{v}\right) = \frac{1}{\alpha (t)} \left( \mathsf h (t) - e^f |\nabla f|^2 \right) \nonumber \\&\le \frac{1}{\alpha (t)} (\mathsf h (t) - \underline{M} |\nabla f|^2). \end{aligned}$$

Let $x_1, x_2 \in \mathscr {M}$ and $0<t_1<t_2<T$. Let $\gamma \in \mathscr {C}^1( [t_1,t_2]; \mathscr {M})$ be a shortest geodesic curve satisfying $\gamma (t_1) = x_1$, $\gamma (t_2) = x_2$ and $|\dot{\gamma }| = d(x_2, x_1)/(t_2 -t_1)$ where $\dot{\gamma }= d\gamma /dt$. Then using (5.3) it is seen that

5.4 $$\begin{aligned} f(x_1, t_1) - f(x_2, t_2)&= \int _{t_2}^{t_1}\frac{d}{dt} [f(\gamma (t),t)] \, dt = \int _{t_2}^{t_1} [\langle \nabla f(\gamma (t),t), \dot{\gamma }(t) \rangle + \partial _t f(\gamma (t),t)] \, dt\nonumber \\&= \int _{t_1}^{t_2} [-\langle \nabla f(\gamma (t),t), \dot{\gamma }(t) \rangle - \partial _t f(\gamma (t),t)] \, dt\nonumber \\&\le \int _{t_1}^{t_2} \left( |\nabla f(\gamma (t),t)|| \dot{\gamma }(t)| +\frac{1}{\alpha (t)} [\mathsf h (t) -\underline{M} |\nabla f (\gamma (t),t)|^2] \right) \, dt \nonumber \\&\le \int _{t_1}^{t_2} \left( -\frac{\underline{M}}{\alpha (t)} |\nabla f(\gamma (t),t)|^2 + |\nabla f(\gamma (t),t)|| \dot{\gamma }(t)|\right) \, dt + \int _{t_1}^{t_2} \frac{\mathsf h (t)}{\alpha (t)} \, dt \nonumber \\&\le \int _{t_1}^{t_2} \frac{| \dot{\gamma }(t)|^2}{4 \underline{M}} \alpha (t) \, dt + \int _{t_1}^{t_2} \frac{\mathsf h (t)}{\alpha (t)} \, dt, \end{aligned}$$

where we have used $-\mathsf a x^2 + \mathsf b x \le \mathsf b^2 /(4 \mathsf a)$. Note that in the event $\underline{M}=0$ by interpretting $1/\underline{M}$ as infinity the inequalities (5.4) and (5.5) below are trivially true. Exponentiating the above inequality and substituting for $|\dot{\gamma }|$ gives

5.5 $$\begin{aligned} v(x_1, t_1) \le v(x_2, t_2)~ \textrm{exp} \left[ \frac{d^2(x_2, x_1)}{(t_2-t_1)^2} \int _{t_1}^{t_2} \frac{\alpha (t)}{4\underline{M}} \, dt + \int _{t_1}^{t_2} \frac{\mathsf h (t)}{\alpha (t)} \, dt\right] . \end{aligned}$$

To complete the proof of the Harnack inequality (2.10) it suffices to use (2.9) to write

5.6 $$\begin{aligned} {\mathsf h}(t)&= \beta (t) + \alpha (t) \sup _{\mathscr {M} \times [0, T]} [-\mathscr {G}/v]_+ + b \alpha ^2(t) \sup _{\mathscr {M} \times [0, T]} [\mathscr {G}_v+\mathsf L] _+ \nonumber \\&+ \alpha (t) \sqrt{b} \sup _{\mathscr {M} \times [0, T]} \bigg [ \frac{b \alpha ^2 (\alpha -1)^2 }{4(\alpha -1)^2- 2\varepsilon b \alpha ^2} \bigg [ \frac{\mathscr {G}}{v} - \mathscr {G}_v - \frac{\alpha (p-1)}{\alpha -1} v\mathscr {G}_{vv} +\frac{(\alpha -1)}{b \alpha ^2} \beta \bigg ]_+^2 \nonumber \\&+\beta \mathscr {G}_v -\alpha (p-1) \Delta _\phi \mathscr {G}^x + \frac{3v^{2/3}}{2\varepsilon ^{1/3}} \left[ (\alpha -1) \frac{|\mathscr {G}_x|}{v}+ \alpha (p-1) |\mathscr {G}_{x v}| \right] ^{4/3} + \mathsf M\bigg ] _+^{1/2}, \end{aligned}$$

and so

5.7 $$\begin{aligned} \frac{{\mathsf h}(t)}{\alpha (t)}&= \frac{\beta (t)}{\alpha (t)} + \sup _{\mathscr {M} \times [0, T]} [-\mathscr {G}/v]_+ + b \alpha (t) \sup _{\mathscr {M} \times [0, T]} [\mathscr {G}_v+\mathsf L] _+ \nonumber \\&\quad + \sqrt{b} \sup _{\mathscr {M} \times [0, T]} \bigg [ \frac{b \alpha ^2 (\alpha -1)^2 }{4(\alpha -1)^2- 2\varepsilon b \alpha ^2} \bigg [ \frac{\mathscr {G}}{v} - \mathscr {G}_v - \frac{\alpha (p-1)}{\alpha -1} v\mathscr {G}_{vv} +\frac{(\alpha -1)}{b \alpha ^2} \beta \bigg ]_+^2 \nonumber \\&\quad +\beta \mathscr {G}_v -\alpha (p-1) \Delta _\phi \mathscr {G}^x + \frac{3v^{2/3}}{2\varepsilon ^{1/3}} \left[ (\alpha -1) \frac{|\mathscr {G}_x|}{v}+ \alpha (p-1) |\mathscr {G}_{x v}| \right] ^{4/3} + \mathsf M\bigg ] _+^{1/2} \nonumber \\&\le \frac{\beta (t)}{\alpha (t)} + {\mathsf H}, \end{aligned}$$

where $\mathsf H$ is as in (2.11). Note that in view of $\alpha$ being nondecreasing we have $\alpha (T) = \sup \alpha$ over [0, *T*]. Substitution in (5.5) gives the desired conclusion. $\square$

### Proof of Theorem 2.6

The idea is to use (2.10) in Theorem 2.3, substitute for the functions $\alpha (t)$ and $\beta (t)$ from (2.20)–(2.21) and integrate. Towards this end, according to (2.10) we have

5.8 $$\begin{aligned} \frac{v(x_1, t_1)}{v(x_2, t_2)} \le \textrm{exp} \left[ \frac{d^2(x_2, x_1)}{(t_2-t_1)^2} \int _{t_1}^{t_2} \frac{\alpha (t)}{4\underline{M}} \, dt + \int _{t_1}^{t_2} \frac{\beta (t)}{\alpha (t)} \, dt + {\mathsf H}(t_2-t_1) \right] . \end{aligned}$$

Referring to (2.20)–(2.21) and the right-hand side of (5.8) it can be seen that

5.9 $$\begin{aligned} \int _{t_1}^{t_2} \alpha (t) \, dt&= \int _{t_1}^{t_2} \left[ 1+\frac{\cosh (MKt) \sinh (MKt)-MKt}{\sinh ^2(MKt)} \right] dt\nonumber \\&= \left[ t + \frac{MKt \coth (MKt)-1}{MK} \right] _{t_1}^{t_2}, \end{aligned}$$

and

5.10 $$\begin{aligned} \int _{t_1}^{t_2} \frac{\beta (t)}{\alpha (t)} \, dt&= \int _{t_1}^{t_2} \frac{b MK \sinh ^2(MKt) [ \coth (MKt) +1] \, dt }{\sinh ^2(MKt)+\cosh (MKt) \sinh (MKt)-MKt} \nonumber \\&= \left[ \frac{b}{2} \log \frac{\sinh (2MKt) + \cosh (2MKt) - 2MKt-1}{2MK}\right] _{t_1}^{t_2}, \end{aligned}$$

The conclusion now follows by substituting these for the integrals in (5.8) and using the bound universal bound $\alpha (T) \le 2$ in (2.11) and exponentiating. $\square$

## Proof of the second local estimate in Theorem 2.8

### Lemma 6.1

Under the assumptions of Lemma 3.3, if ${\mathscr {R}ic}_\phi ^m(g) \ge -(m-1) k g$ and $\alpha (t) \ge 1$ satisfies $1+MKt -t\alpha '/2=\alpha$, where $M=\sup v$ and $K=(p-1)(m-1) k$, then we have

6.1 $$\begin{aligned} \mathscr {L} [F] \le&- \frac{1}{b\alpha ^2} F^2 - \frac{1}{b\alpha ^2} \left[ (\alpha -1)\frac{|\nabla v|^2}{v} +\beta -\frac{b \alpha }{t} (1+MKt)\right] ^2\nonumber \\&- \frac{2F}{b\alpha ^2} \left[ (\alpha -1)\frac{|\nabla v|^2}{v} +\beta -\frac{b \alpha }{t}(1+MKt)\right] -\frac{2}{t}F+\mathscr {G}_v F\nonumber \\&+ 2p \langle \nabla F, \nabla v \rangle -\frac{2}{t} \beta +\frac{b}{t^2} (1+MKt)^2 -\beta ' + \mathscr {G}_v \beta \nonumber \\&+\left[ (\alpha -1) \left( \frac{\mathscr {G}}{v} -\mathscr {G}_v\right) - \alpha (p-1)v \mathscr {G}_{vv}\right] \frac{|\nabla v|^2}{v}\nonumber \\&- \alpha (p-1) \Delta _ \phi \mathscr {G}^x +\left[ 2 (\alpha -1)\frac{|\mathscr {G}_x|}{v} + 2\alpha (p-1) |\mathscr {G}_{x v}|\right] |\nabla v|. \end{aligned}$$

### Proof

Starting from (3.9), using the curvature lower bound ${\mathscr {R}ic}_\phi ^m (g) \ge -(m-1)k g$, $0<v\le M$ and setting $K=(p-1)(m-1) k$ we can write

6.2 $$\begin{aligned} \mathscr {L} [F] \le&- \frac{1}{b}[(p-1)\Delta _\phi v]^2+2M K \frac{|\nabla v|^2}{v} + 2p \langle \nabla F, \nabla v \rangle \nonumber \\&+\frac{2}{v}(1-\alpha ) \langle \nabla \mathscr {G}, \nabla v \rangle - \alpha (p-1) \Delta _\phi \mathscr {G} +(\alpha -1) \frac{|\nabla v|^2}{v} \frac{\mathscr {G}}{v} \nonumber \\&+ \alpha ' \frac{\mathscr {G}}{v} -\alpha ' \frac{\partial _t v}{v} -\beta ' - (\alpha -1) \left[ \frac{\partial _t v}{v} - \frac{\mathscr {G}}{v} \right] ^2, \end{aligned}$$

or by adding and subtracting $b(1/t+MK)$ inside the squared bracket term that

6.3 $$\begin{aligned} \mathscr {L} [F] \le&- \frac{1}{b} \left[ (p-1)\Delta _\phi v+ \frac{b}{t} (1+MKt) \right] ^2 + \frac{2}{t} (1+MKt) (p-1)\Delta _\phi v\nonumber \\&+ \frac{b}{t^2} (1+MKt)^2+2M K \frac{|\nabla v|^2}{v} + 2p \langle \nabla F, \nabla v \rangle -\alpha ' \frac{\partial _t v}{v}-\beta ' \nonumber \\&-(\alpha -1) \left[ \frac{\partial _t v}{v} - \frac{\mathscr {G}}{v} \right] ^2 +\frac{2}{v}(1-\alpha ) \langle \nabla \mathscr {G}, \nabla v \rangle - \alpha (p-1) \Delta _\phi \mathscr {G} \nonumber \\&+(\alpha -1) \frac{|\nabla v|^2}{v} \frac{\mathscr {G}}{v} + \alpha ' \frac{\mathscr {G}}{v}. \end{aligned}$$

Substituting from (3.2) gives

6.4 $$\begin{aligned} \mathscr {L} [F] \le&- \frac{1}{b}\left[ (p-1)\Delta _\phi v+ \frac{b}{t} (1+MKt)\right] ^2 + \frac{2}{t} (1+MKt) \left[ \frac{\partial _t v}{v}-\frac{|\nabla v|^2}{v} -\frac{\mathscr {G}}{v}\right] \nonumber \\&+ \frac{b}{t^2} (1+MKt)^2+2MK \frac{|\nabla v|^2}{v} + 2p \langle \nabla F, \nabla v \rangle -\alpha ' \frac{\partial _t v}{v}-\beta ' \nonumber \\&-(\alpha -1) \left[ \frac{\partial _t v}{v} - \frac{\mathscr {G}}{v} \right] ^2 +\frac{2}{v}(1-\alpha ) \langle \nabla \mathscr {G}, \nabla v \rangle - \alpha (p-1) \Delta _\phi \mathscr {G} \nonumber \\&+(\alpha -1) \frac{|\nabla v|^2}{v} \frac{\mathscr {G}}{v} + \alpha ' \frac{\mathscr {G}}{v}, \end{aligned}$$

or after rearranging terms that

6.5 $$\begin{aligned} \mathscr {L} [F] \le&- \frac{1}{b}\left[ (p-1)\Delta _\phi v+ \frac{b}{t} (1+MKt)\right] ^2\nonumber \\&-\frac{2}{t} \left[ \frac{|\nabla v|^2}{v} - \frac{2(1+MKt)-t\alpha '}{2}\frac{\partial _t v}{v} + \frac{2(1+MKt)-t\alpha '}{2}\frac{\mathscr {G}}{v}-\beta \right] \nonumber \\&-\frac{2}{t} \beta + \frac{b}{t^2} (1+MKt)^2+ 2p \langle \nabla F, \nabla v \rangle -\beta ' -(\alpha -1) \left[ \frac{\partial _t v}{v} - \frac{\mathscr {G}}{v} \right] ^2\nonumber \\&+\frac{2}{v}(1-\alpha ) \langle \nabla \mathscr {G}, \nabla v \rangle - \alpha (p-1) \Delta _\phi \mathscr {G}+(\alpha -1) \frac{|\nabla v|^2}{v} \frac{\mathscr {G}}{v}. \end{aligned}$$

Let us now suppose that the function $\alpha$ is chosen such that the following holds

6.6 $$\begin{aligned} 1+MKt -t\alpha '/2=\alpha . \end{aligned}$$

Then using this identity along with

6.7 $$\begin{aligned} (2/v)(1-\alpha ) \langle&\nabla v, \nabla \mathscr {G} \rangle - \alpha (p-1) \Delta _\phi \mathscr {G} \nonumber \\ =&~ (2/v) \langle \nabla v, (1-\alpha ) \mathscr {G}_x -\alpha (p-1) v\mathscr {G}_{xv} \rangle + (2/v) (1-\alpha ) \mathscr {G}_v |\nabla v|^2 \nonumber \\&- \alpha (p-1) [\Delta _ \phi \mathscr {G}^x + \mathscr {G}_{vv} |\nabla v|^2+ \mathscr {G}_v \Delta _\phi v]. \end{aligned}$$

and discarding the negative term $-(\alpha -1)[\partial _t v -\mathscr {G}]^2/v^2$ we can rewrite (6.5) as

6.8 $$\begin{aligned} \mathscr {L} [F] \le&- \frac{1}{b}\left[ (p-1)\Delta _\phi v+\frac{b}{t} ( 1+MKt)\right] ^2 -\frac{2}{t}F+ 2p \langle \nabla F, \nabla v \rangle \nonumber \\&-\frac{2}{t} \beta +\frac{b}{t^2}(1+MKt)^2 -\beta ' - \alpha (p-1) \mathscr {G}_v \Delta _\phi v\nonumber \\&+\left[ (\alpha -1) \left( \frac{\mathscr {G}}{v} -2\mathscr {G}_v\right) - \alpha (p-1)v \mathscr {G}_{vv}\right] \frac{|\nabla v|^2}{v}\nonumber \\&- \alpha (p-1) \Delta _ \phi \mathscr {G}^x +\frac{2}{v} \langle \nabla v, (1-\alpha ) \mathscr {G}_x -\alpha (p-1) v\mathscr {G}_{xv} \rangle . \end{aligned}$$

Next, by recalling (3.2) and (3.7) we have $(p-1)\Delta _\phi v =- [F + (\alpha -1)|\nabla v|^2/v + \beta ]/\alpha$ and

6.9 $$\begin{aligned} (p-1)\Delta _\phi v + \frac{b}{t} (1 +MKt)&= [\partial _t v -|\nabla v|^2 - \mathscr {G}]/v+\frac{b}{t} (1 +MKt)\nonumber \\&=-\frac{1}{\alpha } \left[ F + (\alpha -1)\frac{|\nabla v|^2}{v} +\beta -\frac{b \alpha }{t} (1+MKt)\right] . \end{aligned}$$

Substituting both these identities in (6.8) gives

6.10 $$\begin{aligned} \mathscr {L} [F] \le&- \frac{1}{b\alpha ^2}\left[ F + (\alpha -1)\frac{|\nabla v|^2}{v} +\beta -\frac{b \alpha }{t} (1+MKt)\right] ^2-\frac{2}{t}F \nonumber \\&+ 2p \langle \nabla F, \nabla v \rangle -\frac{2}{t} \beta +\frac{b}{t^2} (1+MKt)^2 -\beta ' + \mathscr {G}_v [F + \beta ]\nonumber \\&+\left[ (\alpha -1) \left( \frac{\mathscr {G}}{v} -\mathscr {G}_v\right) - \alpha (p-1)v \mathscr {G}_{vv}\right] \frac{|\nabla v|^2}{v}\nonumber \\&- \alpha (p-1) \Delta _ \phi \mathscr {G}^x +\frac{2}{v} \langle \nabla v, (1-\alpha ) \mathscr {G}_x -\alpha (p-1) v\mathscr {G}_{xv} \rangle . \end{aligned}$$

Expanding the squared term on the first line and using (4.7) to bound the second term on the last line gives the desired conclusion. $\square$

Consider $G = \gamma \eta F$ where $\gamma =\gamma (t) \ge 0$ (with $\gamma (t)>0$ for $t>0$ and $\gamma (0)=0$) is to be specified below, $\eta =\eta (x)$ is the cut-off function in (3.26) and *F* is as in (3.7). Let $(x_1,t_1)$ denote the point where *G* attains its maximum over $Q_{2R, T_1}$. We assume $G (x_1, t_1)>0$ as otherwise the estimate immediately follows from $G= \gamma \eta F \le 0$. So in particular $t_1>0$ and from (*ii*) in Lemma 3.4 we have $\varrho (x_1) < 2R$. Therefore at $(x_1, t_1)$ it holds $\partial _t G \ge 0$, $\nabla G =0$, $\Delta _\phi G \le 0$ and so $\mathscr {L} [G] \ge 0$. Substituting from (6.1) gives

6.11 $$\begin{aligned} 0 \le \mathscr {L} [G] =&~ \gamma \eta \mathscr {L} [F] +2 \gamma (p-1) v(|\nabla \eta |^2/\eta ) F - \gamma F (p-1) v \Delta _\phi \eta +\gamma ' \eta F\nonumber \\ =&- \frac{\gamma \eta }{b\alpha ^2} F^2 - \frac{\gamma \eta }{b\alpha ^2} \left[ (\alpha -1)\frac{|\nabla v|^2}{v} +\beta -\frac{b \alpha }{t_1}(1+MKt_1)\right] ^2\nonumber \\&- \frac{2 \gamma \eta F}{b\alpha ^2} \left[ (\alpha -1)\frac{|\nabla v|^2}{v} \!+\! \beta -\frac{b \alpha }{t_1} (1+MK t_1)\right] \!-\! \frac{2}{t_1} \gamma \eta F+\mathscr {G}_v \gamma \eta F\nonumber \\&+ 2p \gamma \eta \langle \nabla F, \nabla v \rangle + \left[ -\frac{2}{t_1} \beta +\frac{b}{t_1^2} ( 1+MKt_1)^2 -\beta ' + \mathscr {G}_v \beta \right] \gamma \eta \nonumber \\&- \alpha (p-1) \gamma \eta \Delta _ \phi \mathscr {G}^x +\left[ 2 (\alpha -1)\frac{|\mathscr {G}_x|}{v} + 2\alpha (p-1) |\mathscr {G}_{x v}|\right] \gamma \eta |\nabla v|\nonumber \\&+\left[ (\alpha -1) \left( \frac{\mathscr {G}}{v} -\mathscr {G}_v\right) - \alpha (p-1)v \mathscr {G}_{vv}\right] \gamma \eta \frac{|\nabla v|^2}{v} \nonumber \\&+2 \gamma (p-1) v\frac{|\nabla \eta |^2}{\eta } F - \gamma F (p-1) v \Delta _\phi \eta +\gamma ' \eta F. \end{aligned}$$

By making use of $\nabla G= \gamma \nabla (\eta F)=0$ and so $\eta \nabla F= -F \nabla \eta$ at $(x_1, t_1)$ and rewriting the right-hand side in terms of *G* we have

6.12 $$\begin{aligned} 0 \le&- \frac{G^2}{b\alpha ^2 \gamma \eta } - \frac{\gamma \eta (\alpha -1)^2}{b\alpha ^2} \frac{|\nabla v|^4}{v^2} - \frac{\gamma \eta }{b\alpha ^2} \left[ \beta -\frac{b \alpha }{t_1} (1+MK t_1)\right] ^2\nonumber \\&- \frac{2\gamma \eta (\alpha -1)}{b\alpha ^2} \left[ \beta -\frac{b \alpha }{t_1} (1+MKt_1)\right] \frac{|\nabla v|^2}{v} -\frac{2}{t_1} G+\mathscr {G}_v G\nonumber \\&- \frac{2 G}{b\alpha ^2} \left[ (\alpha -1)\frac{|\nabla v|^2}{v} +\beta - \frac{b \alpha }{t_1} (1+MKt_1)\right] - 2p \nabla v \frac{\nabla \eta }{\eta } G\nonumber \\&+ \left[ -\frac{2}{t_1} \beta +\frac{b}{t_1^2} (1+MKt_1)^2 -\beta ' + \mathscr {G}_v \beta - \alpha (p-1) \Delta _ \phi \mathscr {G}^x \right] \gamma \eta \nonumber \\&+\left[ (\alpha -1) \left( \frac{\mathscr {G}}{v} -\mathscr {G}_v\right) - \alpha (p-1)v \mathscr {G}_{vv}\right] \gamma \eta \frac{|\nabla v|^2}{v} \nonumber \\&+\left[ 2 (\alpha -1)\frac{|\mathscr {G}_x|}{v} + 2\alpha (p-1) |\mathscr {G}_{x v}|\right] \gamma \eta |\nabla v|\nonumber \\&+2 (p-1) v\frac{|\nabla \eta |^2}{\eta ^2} G - (p-1) v \frac{\Delta _\phi \eta }{\eta } G +\frac{\gamma '}{\gamma } G. \end{aligned}$$

Rearranging terms and multiplying through by $\eta$ gives

6.13 $$\begin{aligned} 0&\le - \frac{G^2}{b\alpha ^2 \gamma } - \frac{(\alpha -1)^2}{b\alpha ^2}\gamma \eta ^2 \frac{|\nabla v|^4}{v^2} -\frac{2 (\alpha -1)}{b \alpha ^2} \frac{|\nabla v|^2}{v} \eta G - 2 p \nabla v \frac{\nabla \eta }{\sqrt{\eta }} \sqrt{\eta }G\nonumber \\&+\left\{ \frac{\gamma '}{\gamma } - (p-1) v \frac{\Delta _\phi \eta }{\eta } +2 (p-1) v\frac{|\nabla \eta |^2}{\eta ^2} -\frac{2}{t_1} +\mathscr {G}_v - \frac{2}{b\alpha ^2} \left[ \beta - \frac{b \alpha }{t_1} (1+MKt_1)\right] \right\} \eta G\nonumber \\&+ \left\{ \frac{b}{t_1^2} (1+MKt_1)^2 -\frac{2}{t_1} \beta -\beta ' + \mathscr {G}_v \beta - \alpha (p-1) \Delta _ \phi \mathscr {G}^x \right. \nonumber \\&\quad \left. - \frac{1}{b\alpha ^2}\left[ \beta -\frac{b \alpha }{t_1} (1+MKt_1)\right] ^2\right\} \gamma \eta ^2\nonumber \\&+\left\{ (\alpha -1) \left( \frac{\mathscr {G}}{v} -\mathscr {G}_v\right) - \alpha (p-1)v \mathscr {G}_{vv} - \frac{2(\alpha -1)}{b\alpha ^2} \left[ \beta -\frac{b\alpha }{t_1} (1+MKt_1)\right] \right\} \gamma \eta ^2 \frac{|\nabla v|^2}{v}\nonumber \\&+\left[ 2 (\alpha -1)\frac{|\mathscr {G}_x|}{v} + 2\alpha (p-1) |\mathscr {G}_{x v}|\right] \gamma \eta ^2 |\nabla v|. \end{aligned}$$

We now bound the terms on the right-hand side of (6.13). Making use of the inequalities (4.11) and (4.12) as before along with

6.14 $$\begin{aligned}&-\frac{(\alpha -1)^2- \varepsilon b \alpha ^2 }{2 b \alpha ^2} \frac{|\nabla v|^4}{v^2}+\bigg \{(\alpha -1) \left( \frac{\mathscr {G}}{v} -\mathscr {G}_v\right) \nonumber \\&\qquad \qquad - \alpha (p-1)v \mathscr {G}_{vv} - \frac{2(\alpha -1)}{b\alpha ^2} \left[ \beta -\frac{b\alpha }{t_1} (1+MKt_1)\right] \bigg \} \frac{|\nabla v|^2}{v} \nonumber \\&\qquad \qquad \le \frac{b \alpha ^2 (\alpha -1)^2 }{4(\alpha -1)^2- 2\varepsilon b \alpha ^2} \left[ \frac{\mathscr {G}}{v} - \mathscr {G}_v - \frac{\alpha (p-1)}{\alpha -1} v\mathscr {G}_{vv} - \frac{2[\beta t_1-b\alpha (1+MKt_1)]}{b \alpha ^2 t_1} \right] _+^2, \end{aligned}$$

the bounds (3.27)–(3.28) on the cut-off function $\eta$ and $0<v\le M$ and substituting these into (6.13) it follows after multiplying through by $\gamma$ that

6.15 $$\begin{aligned} 0 \le&- \frac{G^2}{b\alpha ^2} + \bigg \{\frac{b \alpha ^2 p^2 M \gamma }{2(\alpha -1)} \frac{c_1 ^2}{R^2} +\frac{(p-1)M \gamma }{R^2} [c_2 +(m-1) c_1(1+R \sqrt{k})+ 2 c_1^2] \nonumber \\&+\gamma \eta \left[ \frac{\gamma '}{\gamma } - \frac{2 [\beta t_1- b \alpha (1+MKt_1)]}{b\alpha ^2 t_1} -\frac{2}{t_1} +\mathscr {G}_v\right] _+ \bigg \} G\nonumber \\&+ \bigg \{\frac{b}{t_1^2} (1+MKt_1)^2 -\frac{2}{t_1} \beta -\beta ' + \mathscr {G}_v \beta - \alpha (p-1) \Delta _ \phi \mathscr {G}^x \nonumber \\&- \frac{[\beta t_1 -b \alpha (1+MKt_1)]^2}{b\alpha ^2t_1^2} + \frac{3v^{2/3}}{2\varepsilon ^{1/3}} \left[ (\alpha -1) \frac{|\mathscr {G}_x|}{v} + \alpha (p-1) |\mathscr {G}_{x v}| \right] ^{4/3} \nonumber \\&+\frac{b \alpha ^2 (\alpha -1)^2 }{4(\alpha -1)^2- 2\varepsilon b \alpha ^2} \left[ \frac{\mathscr {G}}{v} - \mathscr {G}_v - \frac{\alpha (p-1)}{\alpha -1} v\mathscr {G}_{vv} - \frac{2[\beta t_1-b\alpha (1+MKt_1)]}{b \alpha ^2 t_1} \right] _+^2\bigg \}\gamma ^2 \eta ^2. \end{aligned}$$

Let us set $M = \sup v$ where the supremum is taken over $Q_{2R, T}$ and denote by $\textbf{I}$ and $\textbf{II}$ the coefficients of *G* and $\gamma ^2 \eta ^2=\gamma ^2 \eta ^2 G^0$ on the right-hand side of (6.15) respectively. Therefore we have

6.16 $$\begin{aligned} \textbf{I} =&~ \frac{b \alpha ^2 p^2 M \gamma }{2(\alpha -1)} \frac{c_1 ^2}{R^2} +\frac{(p-1)M \gamma }{R^2} [c_2 +(m-1) c_1(1+R \sqrt{k})+ 2 c_1^2] \nonumber \\&+ \gamma \eta \left[ \frac{\gamma '}{\gamma } - \frac{2 [\beta t_1- b \alpha (1+MKt_1)]}{b\alpha ^2 t_1} -\frac{2}{t_1} +\mathscr {G}_v\right] _+, \end{aligned}$$

and

6.17 $$\begin{aligned} \textbf{II} =&~\frac{b}{t_1^2} (1+MKt_1)^2 -\frac{2}{t_1} \beta -\beta ' + \beta \mathscr {G}_v - \alpha (p-1) \Delta _ \phi \mathscr {G}^x\nonumber \\&- \frac{[\beta t_1 -b \alpha (1+MKt_1)]^2}{b\alpha ^2t_1^2} +\frac{3v^{2/3}}{2\varepsilon ^{1/3}} \left[ (\alpha -1) \frac{|\mathscr {G}_x|}{v} + \alpha (p-1) |\mathscr {G}_{x v}| \right] ^{4/3}\nonumber \\&+\frac{b \alpha ^2 (\alpha -1)^2 }{4(\alpha -1)^2- 2\varepsilon b \alpha ^2} \left[ \frac{\mathscr {G}}{v} - \mathscr {G}_v - \frac{\alpha (p-1)}{\alpha -1} v\mathscr {G}_{vv} - \frac{2[\beta t_1-b\alpha (1+MKt_1)]}{b \alpha ^2 t_1} \right] _+^2. \end{aligned}$$

In particular (6.15) can be re-written as $0 \le - G^2/(b \alpha ^2) + \textbf{I} G+ \gamma ^2 \eta ^2 \textbf{II}$. With the aid of the constants and quantities described above let us introduce some further notation6.186.196.20and subsequently using the above,

6.21 $$\begin{aligned} \tilde{\mathsf A} = \overbrace{b \alpha ^2 p^2 M \lambda \frac{c_1 ^2}{R^2}}^{{\mathsf C}={\mathsf C}(t)} + \underbrace{\gamma \left\{ \frac{(p-1)M}{R^2} [c_2 +(m-1) c_1(1+R \sqrt{k}) + 2 c_1^2] + \sup _{Q_{2R,T}} [\mathscr {G}_v+\tilde{\mathsf L}] _+ \right\} }_{\gamma (t) {\tilde{\mathsf D}}}, \end{aligned}$$

and

6.22 $$\begin{aligned} \tilde{\mathsf B}&= \sup _{Q_{2R,T}} \left[ \frac{b \alpha ^2 (\alpha -1)^2 }{4(\alpha -1)^2- 2\varepsilon b \alpha ^2} \bigg [ \frac{\mathscr {G}}{v} - \mathscr {G}_v - \frac{\alpha (p-1)}{\alpha -1} v\mathscr {G}_{vv} - \frac{2[\beta t-b\alpha (1+MKt)]}{b \alpha ^2 t} \right] _+^2 \nonumber \\&+\beta \mathscr {G}_v - \alpha (p-1) \Delta _ \phi \mathscr {G}^x +\frac{3v^{2/3}}{2\varepsilon ^{1/3}} \left[ (\alpha -1) \frac{|\mathscr {G}_x|}{v} + \alpha (p-1) |\mathscr {G}_{x v}| \right] ^{4/3}+\tilde{\mathsf M} \bigg ]_+. \end{aligned}$$

Then in view of (6.16) and (6.17) and upon recalling (2.7) we have $\textbf{I} \le \tilde{\mathsf A}$ and $\textbf{II} \le \tilde{\mathsf B}$ and so (6.15) gives,

6.23 $$\begin{aligned} 0 \le -\frac{1}{b \alpha ^2} G^2 +\tilde{ \mathsf A} G + \gamma ^2 \tilde{\mathsf B}. \end{aligned}$$

We now make use of the implication $-\mathsf a x^2 +\mathsf bx +\mathsf c \ge 0 \implies x \le \mathsf b/\mathsf a +\sqrt{\mathsf c/\mathsf a}$ (with ${\mathsf a}>0$, ${\mathsf b}, {\mathsf c} \ge 0$) to deduce that

6.24 $$\begin{aligned} G(x_1, t_1)&\le b \alpha ^2(t_1) \tilde{\mathsf A} (t_1) + \alpha (t_1) \gamma (t_1) \sqrt{b \tilde{\mathsf B}}\nonumber \\&= b \alpha ^2(t_1)[\mathsf C (t_1) + \gamma (t_1) \tilde{\mathsf D}]+ \alpha (t_1) \gamma (t_1) \sqrt{b \tilde{\mathsf B}}. \end{aligned}$$

Since $\eta \equiv 1$ for $d(x,x_0) \le R$ and $(x_1, t_1)$ is the point where $G= \gamma (t) \eta F$ attains its maximum on $Q_{2R, T_1}$ we have

6.25 $$\begin{aligned} G(x, T_1)= \gamma (T_1) F(x, T_1) = [\gamma \eta F](x, T_1) \le [\gamma \eta F](x_1, t_1) = G(x_1, t_1). \end{aligned}$$

Hence making note of the bound (6.24) and dividing through by $T_1 >0$ gives

6.26 $$\begin{aligned} F(x,T_1) \le \frac{1}{\gamma (T_1)} G(x_1, t_1)&\le b \alpha ^2(t_1)\left[ \frac{\mathsf C (t_1)}{\gamma (T_1)} + \frac{\gamma (t_1)}{\gamma (T_1)} \tilde{\mathsf D}\right] + \alpha (t_1) \frac{\gamma (t_1)}{\gamma (T_1)} \sqrt{b \tilde{\mathsf B}}, \end{aligned}$$

and so in view of $1< \alpha (t_1)\le \alpha (T_1)$ and $0< \gamma (t_1) \le \gamma (T_1)$ due to the monotonicity of $\alpha$ and $\gamma$ this gives

6.27 $$\begin{aligned} F(x,T_1)&\le b \alpha ^2(t_1) \frac{\mathsf C(t_1)}{\gamma (T_1)} +\frac{\gamma (t_1)}{\gamma (T_1)} [b \alpha ^2(t_1) \tilde{\mathsf D} + \alpha (t_1) \sqrt{b \tilde{\mathsf B}}] \nonumber \\&\le b \alpha ^2(T_1) \frac{\mathsf C(T_1)}{\gamma (T_1)} +b \alpha ^2(T_1) \tilde{\mathsf D} + \alpha (T_1) \sqrt{b \tilde{\mathsf B}}. \end{aligned}$$

The arbitrariness of $T_1 >0$ gives the desired conclusion for any $0< t \le T$. $\square$

## Proof of the Harnack inequality in Theorem 2.10 and Theorem 2.13

In this section we present the proof of the Harnack inequalities resulting from the second gradient estimates in Theorem 2.8 and Theorem 2.12.

### Proof of Theorem 2.10

This is similar to the proof of Theorem 2.3 in Section 5. The only modification is that in (5.5) we use the global estimate (2.33) in Theorem 2.9 which will then result in a different $\mathsf h(t)$ and hence $\mathsf H$ as given in (2.35). $\square$

### Proof of Theorem 2.13

We use (2.34) in Theorem 2.10, substitute for the functions $\alpha (t)$ and $\beta (t)$ from (2.44)–(2.45) and integrate. Indeed referring to (2.44)–(2.45) it can be seen that

7.1 $$\begin{aligned} \int _{t_1}^{t_2} \alpha (t) \, dt&= \int _{t_1}^{t_2} \left[ 1+ \frac{2}{3} MKt\right] dt = \left[ t + \frac{1}{3} MKt^2\right] _{t_1}^{t_2}, \end{aligned}$$

and

7.2 $$\begin{aligned} \int _{t_1}^{t_2} \frac{\beta (t)}{\alpha (t)} \, dt&= \int _{t_1}^{t_2} \frac{[b + b MKt+ \frac{b}{3} (MK)^2t^2] \, dt }{t+ \frac{2}{3} MKt^2} \nonumber \\&= \left[ b \log t - \frac{b}{4} \log (1+\frac{2}{3} MKt) + \frac{b}{2} MKt\right] _{t_1}^{t_2}. \end{aligned}$$

The conclusion now follows by substituting in

7.3 $$\begin{aligned} \frac{v(x_1, t_1)}{v(x_2, t_2)} \le \textrm{exp} \left[ \frac{d^2(x_2, x_1)}{(t_2-t_1)^2} \int _{t_1}^{t_2} \frac{\alpha (t)}{4\underline{M}} \, dt + \int _{t_1}^{t_2} \frac{\beta (t)}{\alpha (t)} \, dt + {\mathsf H} (t_2-t_1) \right] , \end{aligned}$$

and exponentiating accordingly. $\square$

## Proof of the global gradient bounds in Theorem 2.15 and Theorem 2.16

Throughout this section we write $\square = {\mathscr {L}} - \varepsilon \langle \nabla v, \nabla \cdot \rangle$ where $\mathscr {L}$ is the evolution operator in (3.1) with $p>1$, $v=v(x,t)$ and $\varepsilon \in {\mathbb {R}}$ is a fixed constant.

### Lemma 8.1

Let $(\mathscr {M},g,d\mu )$ be a smooth metric measure space with $\mathscr {M}$ closed and $d\mu =e^{-\phi } dv_g$. Let *u* be a positive smooth solution to (1.3) and set $v =pu^{p-1}/(p-1)$ where $p>1$. For $\beta \in \mathbb {R}$, $\zeta =\zeta (t)$ a non-negative, smooth function and $\Gamma =\Gamma (v)$ a given function of class $\mathscr {C}^2(0, \infty )$ set

8.1 $$\begin{aligned} {\mathsf F}[v] = \zeta (t) \frac{|\nabla v|^2}{v^\beta } + \Gamma (v), \qquad x \in {\mathscr {M}}, t \ge 0. \end{aligned}$$

Then ${\mathsf F}$ satisfies the evolution identity

8.2 $$\begin{aligned} \square {\mathsf F} =&~\zeta '(t) \frac{|\nabla v|^2}{v^\beta } - 2 \frac{\zeta (t)}{v^\beta } (p-1) v {\mathscr {R}ic}_\phi ^m (g) (\nabla v, \nabla v)\nonumber \\&+ 2(p-1) \frac{\zeta (t)}{v^\beta } \left[ |\nabla v|^2 \Delta _\phi v - v |\nabla \nabla v|^2 - v \frac{\langle \nabla \phi , \nabla v \rangle ^2}{(m-n)} \right] \nonumber \\&-2\left[ \varepsilon - 2 (\beta (p-1)+1) \right] \zeta (t) \frac{[\nabla \nabla v]}{v^\beta } (\nabla v, \nabla v) + \Gamma _v(v) \mathscr {G}(t,x,v) \nonumber \\&-\beta [\beta (p-1)+p-\varepsilon ] \zeta (t) \frac{|\nabla v|^4}{v^{\beta +1}} + (1-\varepsilon ) \Gamma _v(v) |\nabla v|^2 \nonumber \\&-\beta \frac{\zeta (t)}{v^{\beta +1}} \mathscr {G}(t,x,v) |\nabla v|^2 - (p-1) v \Gamma _{vv}(v) |\nabla v|^2 \nonumber \\&+ 2\frac{\zeta (t)}{v^\beta } \mathscr {G}_v (t,x,v) |\nabla v |^2 + 2 \frac{\zeta (t)}{v^\beta }\langle \nabla v, \mathscr {G}_x(t,x,v) \rangle . \end{aligned}$$

### Proof

As $\square {\mathsf F} = {\mathscr {L}} ({\mathsf F}) - \varepsilon \langle \nabla v, \nabla {\mathsf F} \rangle$ we justify (8.2) by calculating ${\mathscr {L}} ({\mathsf F})$ and $\langle \nabla v, \nabla {\mathsf F} \rangle$ separately. First, by referring to (3.1) and (8.1), and by using linearity, we shall proceed by calculating the fragments ${\mathscr {L}} [|\nabla v|^2/v^\beta ]$ and ${\mathscr {L}} [\Gamma (v)]$ below before putting them together in (8.5).

- For the first fragment we have
  8.3 $$\begin{aligned} {\mathscr {L}} \left[ \frac{|\nabla v|^2}{v^\beta } \right] =&- 2 (p-1) v {\mathscr {R}ic}_\phi ^m(g) \frac{( \nabla v, \nabla v)}{v^\beta } \nonumber \\&+\frac{2 (p-1)}{v^\beta } \left[ |\nabla v|^2 \Delta _\phi v - v |\nabla \nabla v|^2 - v \frac{\langle \nabla \phi , \nabla v \rangle ^2}{(m-n)} \right] \nonumber \\&+ \frac{2[\beta (p-1)+1]}{v^{\beta }}\langle \nabla |\nabla v|^2 , \nabla v\rangle - \beta [\beta (p-1) +p] \frac{|\nabla v|^{4}}{v^{\beta +1}}\nonumber \\&+ \frac{2}{v^\beta }\langle \nabla v, \nabla \mathscr {G}(t,x,v) \rangle - \beta \mathscr {G}(t,x,v) \frac{|\nabla v|^2}{v^{\beta +1}}. \end{aligned}$$
- For the second fragment, by directly differentiating $\Gamma$, we have in turn $\nabla \Gamma (v) = \Gamma _v(v) \nabla v$ and $\Delta _\phi \Gamma (v) = \Gamma _{vv}(v) |\nabla v|^2 + \Gamma _v (v) \Delta _\phi v$, and subsequently
  8.4 $$\begin{aligned} {\mathscr {L}} [\Gamma (v)] =&~\Gamma _v(v) [\partial _t - (p-1) v \Delta _\phi ] (v) - (p-1)v \Gamma _{vv}(v) |\nabla v|^2 \nonumber \\ =&~\Gamma _v(v) [|\nabla v|^2+\mathscr {G} (t,x,v)] - (p-1)v\Gamma _{vv}(v) |\nabla v|^2. \end{aligned}$$

Putting together the above we can write

8.5 $$\begin{aligned} {\mathscr {L}} ({\mathsf F}) =&~ \zeta '(t) \frac{|\nabla v|^2}{v^\beta } + \zeta (t) {\mathscr {L}} \left[ \frac{|\nabla v|^2}{v^\beta } \right] + {\mathscr {L}} [\Gamma (v)] \nonumber \\ =&~ \zeta '(t) \frac{|\nabla v|^2}{v^\beta } - 2\frac{\zeta (t)}{v^\beta } (p-1) v {\mathscr {R}ic}_\phi ^m(g) (\nabla v, \nabla v) \nonumber \\&+ 2(p-1)\frac{\zeta (t)}{v^\beta } \left[ |\nabla v|^2 \Delta _\phi v - v |\nabla \nabla v|^2 - v \frac{\langle \nabla \phi , \nabla v \rangle ^2}{(m-n)} \right] \nonumber \\&+ 2[\beta (p-1)+1] \frac{\zeta (t)}{v^\beta }\langle \nabla |\nabla v|^2 , \nabla v\rangle - \beta [\beta (p-1) +p] \zeta (t) \frac{|\nabla v|^{4}}{v^{\beta +1}}\nonumber \\&+ 2 \frac{\zeta (t)}{v^\beta }\langle \nabla v, \nabla \mathscr {G}(t,x,v) \rangle - \beta \frac{\zeta (t)}{v^{\beta +1}} \mathscr {G} (t,x,v) |\nabla v|^2 \nonumber \\&+ \Gamma _v(v) [|\nabla v|^2+\mathscr {G}(t,x,v)] - (p-1)v\Gamma _{vv}(v) |\nabla v|^2. \end{aligned}$$

Next, using the identities $\langle \nabla v, \nabla (|\nabla v|^2/v^\beta ) \rangle = \langle \nabla v, \nabla |\nabla v|^2 \rangle /v^\beta - \beta |\nabla v|^4/v^{\beta +1}$, and $\langle \nabla v, \nabla \Gamma (v) \rangle = \Gamma _v(v) |\nabla v|^2$ and referring again to (8.1), we can write

8.6 $$\begin{aligned} \langle \nabla v, \nabla {\mathsf F} \rangle =&~\zeta (t) \langle \nabla v, \nabla (|\nabla v|^2/v^\beta ) \rangle + \langle \nabla v, \nabla \Gamma (v) \rangle \nonumber \\ =&~\zeta (t) \langle \nabla v, \nabla |\nabla v|^2 \rangle /v^\beta - \beta \zeta (t) |\nabla v|^4/v^{\beta +1} + \Gamma _v(v) |\nabla v|^2. \end{aligned}$$

Therefore by putting together the above using $\square {\mathsf F} = {\mathscr {L}} ({\mathsf F}) - \varepsilon \langle \nabla v, \nabla {\mathsf F} \rangle$ and substituting from (8.5) and (8.6) this gives

8.7 $$\begin{aligned} \square {\mathsf F} =&~ \zeta '(t) \frac{|\nabla v|^2}{v^\beta } - 2\frac{\zeta (t)}{v^\beta } (p-1) v {\mathscr {R}ic}_\phi ^m(g) (\nabla v, \nabla v) \nonumber \\&+ 2(p-1)\frac{\zeta (t)}{v^\beta } \left[ |\nabla v|^2 \Delta _\phi v - v |\nabla \nabla v|^2 - v \frac{\langle \nabla \phi , \nabla v \rangle ^2}{(m-n)} \right] \nonumber \\&- \left[ 2 \varepsilon - 4(\beta (p-1)+1) \right] \frac{\zeta (t)}{v^\beta } [\nabla \nabla v] (\nabla v, \nabla v) \nonumber \\&- \beta [\beta (p-1) +p -\varepsilon ] \zeta (t) \frac{|\nabla v|^{4}}{v^{\beta +1}} + 2 \frac{\zeta (t)}{v^\beta } \mathscr {G}_v(t,x,v) |\nabla v|^2 \nonumber \\&- \beta \frac{\zeta (t)}{v^{\beta +1}} \mathscr {G}(t,x,v) |\nabla v|^2 + \Gamma _v(v) [\mathscr {G}(t,x,v)+(1-\varepsilon )|\nabla v|^2] \nonumber \\&+ 2 \frac{\zeta (t)}{v^\beta }\langle \nabla v, \mathscr {G}_x(t,x,v) \rangle - (p-1)v\Gamma _{vv}(v) |\nabla v|^2. \end{aligned}$$

A rearrangement of terms gives the desired conclusion. $\square$

### Lemma 8.2

Under the assumptions of Lemma 8.1 and the *m*-Ricci curvature lower bound ${\mathscr {R}ic}^m_\phi (g) \ge - (m-1) k g$ on ${\mathscr {M}}$ for some $k \ge 0$ we have

8.8 $$\begin{aligned} \square {\mathsf F} \le&\left[ \zeta '(t) +2 \zeta (t) [MK+\mathscr {G}_v(t,x,v)] - \beta \zeta (t) \frac{\mathscr {G}(t,x,v)}{v} \right] \frac{|\nabla v|^2}{v^\beta } \nonumber \\&+\zeta (t)\frac{\Omega (\beta , \varepsilon )}{2(p-1)} \frac{|\nabla v|^4}{v^{\beta +1}} + \Gamma _v(v) [\mathscr {G}(t,x,v) + (1-\varepsilon ) |\nabla v|^2] \nonumber \\&+ 2 \frac{\zeta (t)}{v^\beta }\langle \nabla v, \mathscr {G}_x(t,x,v) \rangle - (p-1)v\Gamma _{vv}(v) |\nabla v|^2. \end{aligned}$$

Here $$M=\sup v$$, $$K=(p-1)(m-1) k$$ and

8.9 $$\begin{aligned} \Omega (\beta , \varepsilon ) =&~ \{\varepsilon - 2[\beta (p-1)+1] -(p-1)\}^2\nonumber \\&+ (m-1)(p-1)^2 - 2 (p-1) \beta [\beta (p-1) +p-\varepsilon ]. \end{aligned}$$

### Proof

In order to justify the inequality in the lemma, we begin with (8.2) and writing

8.10 $$\begin{aligned} \square {\mathsf F} =&~ \zeta '(t) \frac{|\nabla v|^2}{v^\beta } - 2\frac{\zeta (t)}{v^\beta } (p-1) v {\mathscr {R}ic}_\phi ^m (g) (\nabla v, \nabla v) \nonumber \\&+2(p-1) \zeta (t) \frac{|\nabla v|^2}{v^\beta } [\Delta v- \langle \nabla \phi , \nabla v \rangle ] - \beta \frac{\zeta (t)}{v^{\beta +1}} \mathscr {G}(t,x,v) |\nabla v|^2 \nonumber \\&- \left[ 2 \varepsilon - 4(\beta (p-1)+1) \right] \frac{\zeta (t)}{v^\beta } [\nabla \nabla v] (\nabla v, \nabla v) -2(p-1) \zeta (t) \frac{|\nabla \nabla v|^2}{v^{\beta -1}} \nonumber \\&- 2(p-1) \frac{\zeta (t)}{v^{\beta -1}} \frac{\langle \nabla \phi , \nabla v \rangle ^2}{(m-n)} - \beta [\beta (p-1) +p-\varepsilon ] \zeta (t) \frac{|\nabla v|^{4}}{v^{\beta +1}} \nonumber \\&+ 2\frac{\zeta (t)}{v^\beta } \mathscr {G}_v (t,x,v) |\nabla v|^2 + \Gamma _v(v) [\mathscr {G}(t,x,v)+(1-\varepsilon )|\nabla v|^2]\nonumber \\&+ 2 \frac{\zeta (t)}{v^\beta }\langle \nabla v, \mathscr {G}_x(t,x,v) \rangle - (p-1)v\Gamma _{vv}(v) |\nabla v|^2. \end{aligned}$$

We now make use of the fact that for any non-zero $n \times n$ symmetric matrix *A*, unit *n*-vector *e* and $a, b \in {\mathbb {R}}$ we have the inequality

8.11 $$\begin{aligned} \left[ a \frac{A(e,e)}{|A|} + b \frac{\textrm{tr} A}{|A|} \right] ^2 \le \max _{A \in \textbf{Sym}_n \atop {A \ne 0, |e|=1}} \left[ a \frac{A (e,e)}{|A|} + b \frac{\textrm{tr} A}{|A|} \right] ^2 = (a+b)^2 +(n-1)b^2. \end{aligned}$$

Towards this end we isolate the terms involving the second derivatives of *v* and write

8.12 $$\begin{aligned} \square {\mathsf F} =&~\frac{\zeta (t)}{v^\beta } \bigg [ \overbrace{2 (p-1) (|\nabla v|^2 \Delta v - v|\nabla \nabla v|^2) - \left[ 2 \varepsilon - 4(\beta (p-1)+1) \right] [\nabla \nabla v] (\nabla v, \nabla v)}^{\textbf{I}=-2(p-1)v|A|^2 + |\nabla v|^2 \{2(p-1) \textrm{tr} A - [2 \varepsilon - 4(\beta (p-1)+1)] A(e,e)\} } \bigg ]\nonumber \\&- 2 \frac{\zeta (t)}{v^\beta } (p-1) v {\mathscr {R}ic}_\phi ^m(g) (\nabla v, \nabla v) -\zeta (t) \beta [\beta (p-1) +p-\varepsilon ] \frac{|\nabla v|^{4}}{v^{\beta +1}}\nonumber \\&-2(p-1) \frac{\zeta (t)}{v^{\beta -1}} \left[ \frac{|\nabla v|^2}{v} \langle \nabla \phi , \nabla v \rangle +\frac{\langle \nabla \phi , \nabla v \rangle ^2}{(m-n)}\right] + \frac{2\zeta (t)}{v^\beta } \mathscr {G}_v (t,x,v) |\nabla v|^2\nonumber \\&+\zeta '(t) \frac{|\nabla v|^2}{v^\beta } - \beta \frac{\zeta (t)}{v^{\beta +1}} |\nabla v|^2\mathscr {G}(t,x,v) + \Gamma _v(v) [\mathscr {G}(t,x,v)+(1-\varepsilon )|\nabla v|^2] \nonumber \\&+ 2 \frac{\zeta (t)}{v^\beta }\langle \nabla v, \mathscr {G}_x(t,x,v) \rangle - (p-1)v\Gamma _{vv}(v) |\nabla v|^2. \end{aligned}$$

Next setting $A=\nabla \nabla v$ with $\textrm{tr} A=\Delta v$ and $e = \nabla v/ |\nabla v|$ we can write the expression inside the brackets on the right-hand side of the first line in (8.12) as

8.13 $$\begin{aligned} \textbf{I} =&- 2(p-1) v |A|^2 + |\nabla v|^2 [2(p-1) \textrm{tr} A -[2 \varepsilon - 4(\beta (p-1)+1)] A (e,e)] \nonumber \\ =&-2 (p-1)v \left[ |A| + \frac{|\nabla v|^2/2}{(p-1)v}\left( [\varepsilon - 2(\beta (p-1)+1) ] \frac{A(e,e)}{|A|} -(p-1)\frac{\textrm{tr} A}{|A|}\right) \right] ^2 \nonumber \\&+ \frac{|\nabla v|^4/2}{(p-1)v} \left[ [\varepsilon - 2(\beta (p-1)+1) ] \frac{A(e,e)}{|A|} -(p-1)\frac{\textrm{tr} A}{|A|}\right] ^2 \nonumber \\ \overbrace{\le }^{p>1}&~\frac{|\nabla v|^4/2}{(p-1)v} \left[ [\varepsilon - 2(\beta (p-1)+1) ] \frac{A(e,e)}{|A|} -(p-1)\frac{\textrm{tr} A}{|A|}\right] ^2. \end{aligned}$$

Now using (8.11) with the choices of constants $a= \varepsilon - 2[\beta (p-1)+1]$ and $b = -(p-1)$ and therefore $(a+b)^2 +(n-1)b^2 = [\varepsilon - 2(\beta (p-1)+1) -(p-1)]^2+ (n-1)(p-1)^2$ it follows from the last inequality in (8.13) that

8.14 $$\begin{aligned} \textbf{I}&\le \left( [\varepsilon - 2(\beta (p-1)+1) -(p-1)]^2+ (n-1)(p-1)^2\right) \frac{|\nabla v|^4/2}{(p-1)v}. \end{aligned}$$

As a result substituting this back into (8.12) gives

8.15 $$\begin{aligned} \square {\mathsf F} \le&~\frac{\zeta (t)}{2(p-1)} \bigg [\{\varepsilon - 2[\beta (p-1)+1] -(p-1)\}^2+ (n-1)(p-1)^2 \bigg ] \frac{|\nabla v|^4}{v^{\beta +1}}\nonumber \\&- 2 \frac{\zeta (t)}{v^\beta } (p-1) v {\mathscr {R}ic}_\phi ^m(g) (\nabla v, \nabla v) - \beta [\beta (p-1) +p-\varepsilon ] \zeta (t) \frac{|\nabla v|^{4}}{v^{\beta +1}}\nonumber \\&-2(p-1) \frac{\zeta (t)}{v^{\beta -1}} \left[ \frac{|\nabla v|^2 }{2v} \sqrt{m-n} +\frac{\langle \nabla \phi , \nabla v \rangle }{\sqrt{m-n}}\right] ^2 - (p-1)v\Gamma _{vv}(v) |\nabla v|^2 \nonumber \\&+ (1-\varepsilon ) \Gamma _v(v) |\nabla v|^2 + \frac{(p-1) (m-n)}{2} \zeta (t) \frac{|\nabla v|^4}{v^{\beta +1}} + \Gamma _v(v) \mathscr {G}(t,x,v) \nonumber \\&+\zeta '(t) \frac{|\nabla v|^2}{v^\beta } + 2\frac{\zeta (t)}{v^\beta } \mathscr {G}_v (t,x,v)|\nabla v|^2 - \beta \frac{\zeta (t)}{v^{\beta +1}} |\nabla v|^2\mathscr {G}(t,x,v)\nonumber \\&+ 2 \frac{\zeta (t)}{v^\beta }\langle \nabla v, \mathscr {G}_x(t,x,v) \rangle . \end{aligned}$$

Continuing further with the inequality, discarding the square term with negative coefficient on the third line and rearranging the remaining terms gives

8.16 $$\begin{aligned} \square {\mathsf F} \le&~\frac{\zeta (t)}{2(p-1)} \bigg [ [\varepsilon - 2(\beta (p-1)+1) -(p-1) ]^2\nonumber \\&+ (m-1)(p-1)^2 - 2 (p-1) \beta [\beta (p-1) +p-\varepsilon ] \bigg ] \frac{|\nabla v|^4}{v^{\beta +1}}\nonumber \\&- 2\frac{\zeta (t)}{v^\beta } (p-1) v {\mathscr {R}ic}_\phi ^m(g) (\nabla v, \nabla v) +\zeta '(t) \frac{|\nabla v|^2}{v^\beta } + 2\frac{\zeta (t)}{v^\beta } \mathscr {G}_v(t,x,v)|\nabla v|^2\nonumber \\&+ \Gamma _v(v) \mathscr {G}(t,x,v) + (1-\varepsilon ) \Gamma _v(v) |\nabla v|^2 - (p-1)v\Gamma _{vv}(v) |\nabla v|^2 \nonumber \\&- \beta \frac{\zeta (t)}{v^{\beta +1}} |\nabla v|^2\mathscr {G}(t,x,v) + 2 \frac{\zeta (t)}{v^\beta }\langle \nabla v, \mathscr {G}_x(t,x,v) \rangle . \end{aligned}$$

Using ${\mathscr {R}ic}^m_\phi (g) \ge - (m-1) k g$, substituting $M=\sup v$, $K=(p-1)(m-1) k$, and recalling (8.9) gives the desired conclusion and completes the proof. $\square$

### Lemma 8.3

Under the assumptions of Lemma 8.1 and Lemma 8.2, ${\mathsf F}$ satisfies the evolution inequality

8.17 $$\begin{aligned} \square {\mathsf F} \le&\left\{ \zeta '(t) + \zeta (t) \left[ 2MK + \frac{2(p-1) v {\mathscr {G}}_v(t,x,v) + {\mathscr {G}}(t,x,v)}{(p-1)v} \right] \right\} v^\frac{1}{p-1} |\nabla v|^2 \nonumber \\&+ \frac{(m-1)(p-1)^2-1}{2(p-1)} \zeta (t) v^\frac{2-p}{p-1} |\nabla v|^4 + \Gamma _v(v) \mathscr {G}(t,x,v) \nonumber \\&+ 2\zeta (t)v^\frac{1}{p-1} \langle \nabla v, \mathscr {G}_x(t,x,v) \rangle - (p-1) [\Gamma _v(v) + v\Gamma _{vv}(v)] |\nabla v|^2. \end{aligned}$$

### Proof

Optimising the quadratic function $\Omega (\beta , \varepsilon )$ in (8.9) leads to an optimising pair $(\beta ^\star , \varepsilon ^\star )$ and an optimal value $\Omega ^\star$ where

8.18 $$\begin{aligned} (\beta ^\star , \varepsilon ^\star ) = \left( -\frac{1}{p-1}, p \right) , \qquad \Omega ^\star =\Omega (\beta ^\star , \varepsilon ^\star ) = (m-1)(p-1)^2-1. \end{aligned}$$

Substituting these values back into (8.8) leads to (8.17). $\square$

### Remark 8.4

The coefficient of $\zeta v^{(2-p)/(p-1)}|\nabla v|^4$ in (8.17) given by $\Omega (\beta ^\star , \varepsilon ^\star )/[2(p-1)]$. This quantity plays an important role in the proofs below and it is negative (i.e., $\le 0$) in the slow diffusion range $p>1$ *iff* $p\le 1+1/\sqrt{m-1}$.

### Proof of Theorem 2.15

Using (8.17) and the assumptions on $\Gamma (v)$ and $\mathscr {G}(t,x,v)$ in the theorem we can write for ${\mathsf F}[v] = \zeta (t) v^{1/(p-1)} |\nabla v|^2 + \Gamma (v)$,

8.19 $$\begin{aligned} \square {\mathsf F} \le&\left\{ \zeta '(t) + \zeta (t) \left[ 2 MK + \frac{2(p-1)v{\mathscr {G}}_v(t,x,v) + {\mathscr {G}}(t,x,v)}{(p-1)v} \right] \right\} v^\frac{1}{p-1} |\nabla v|^2 \nonumber \\&+ \frac{(m-1)(p-1)^2-1}{2(p-1)} \zeta (t) v^\frac{2-p}{p-1} |\nabla v|^4 + \Gamma _v(v) \mathscr {G}(t,x,v) \nonumber \\&+ 2\zeta (t)v^\frac{1}{p-1} \langle \nabla v, \mathscr {G}_x(t,x,v) \rangle - (p-1) [\Gamma _v(v) + v\Gamma _{vv}(v)] |\nabla v|^2 \nonumber \\&\le ~[\zeta '(t) + (2MK+a) \zeta (t)] v^\frac{1}{p-1} |\nabla v|^2 + \frac{(m-1)(p-1)^2-1}{2(p-1)} \zeta (t) v^\frac{2-p}{p-1} |\nabla v|^4. \end{aligned}$$

Now $\zeta (t) = e^{-(2MK+a)t}$ is non-negative, smooth and satisfies $\zeta '+(2MK+a)\zeta =0$. Thus by substituting in (8.19) and noting $\Omega ^\star = \Omega (\beta ^\star , \varepsilon ^\star ) \le 0$ we have $\square {\mathsf F} \le 0$. Finally, the weak maximum principle gives ${\mathsf F}[v](x,t) \le \max _{\mathscr {M}} {\mathsf F}[v](x,0)$ and completes the proof. $\square$

### Proof of Theorem 2.16

For ${\mathsf F}[v] = \zeta (t) v^{1/(p-1)} |\nabla v|^2 + cv^{p/(p-1)}$ where we have set $\Gamma (v)=cv^{p/(p-1)}$ and $(\beta , \varepsilon )=(\beta ^\star , \varepsilon ^\star )$ [*see* (8.18)], using Lemma 8.3 we can write,

8.20 $$\begin{aligned} \square {\mathsf F} \le&\left\{ \zeta '(t) + \zeta (t) \left[ 2MK + \frac{2(p-1)v\mathscr {G}_v(t,x,v) + {\mathscr {G}}(t,x,v)}{(p-1)v} \right] \right\} v^\frac{1}{p-1} |\nabla v|^2 \nonumber \\&+ \frac{(m-1)(p-1)^2-1}{2(p-1)} \zeta (t) v^\frac{2-p}{p-1} |\nabla v|^4 + \Gamma _v(v) \mathscr {G}(t,x,v)\nonumber \\&+ 2\zeta (t)v^\frac{1}{p-1} \langle \nabla v, \mathscr {G}_x(t,x,v) \rangle - (p-1) [\Gamma _v(v) + v\Gamma _{vv}(v)] |\nabla v|^2 \nonumber \\ \le&\left\{ \zeta '(t) + 2MK \zeta (t) + \zeta (t) \frac{2(p-1)v{\mathscr {G}}_v(t,x,v) + {\mathscr {G}}(t,x,v)}{(p-1)v} \right\} v^\frac{1}{p-1} |\nabla v|^2 \nonumber \\&+ \frac{(m-1)(p-1)^2-1}{2(p-1)} \zeta (t) v^\frac{2-p}{p-1} |\nabla v|^4 + \frac{cp}{p-1} v^\frac{1}{p-1} \mathscr {G}(t,x,v) \nonumber \\&- \frac{cp^2}{p-1} v^\frac{1}{p-1} |\nabla v|^2 + 2\zeta (t)v^\frac{1}{p-1} \langle \nabla v, \mathscr {G}_x(t,x,v) \rangle . \end{aligned}$$

In particular, with $c=(p-1)/p^2>0$ and the assumptions in the theorem this gives

8.21 $$\begin{aligned} \square {\mathsf F}&\le ~[\zeta '(t) + 2MK \zeta (t) -1] v^\frac{1}{p-1} |\nabla v|^2 + \frac{(m-1)(p-1)^2-1}{2(p-1)} \zeta (t) v^\frac{2-p}{p-1} |\nabla v|^4. \end{aligned}$$

Now when $k \ge 0$ by taking $\zeta (t) = t/(1+2MK t)$ we have $(\zeta ' + 2 MK \zeta -1) \le 0$. Therefore subject to $1<p \le 1+1/\sqrt{m-1}$ we have $\square {\mathsf F} \le 0$. The conclusion now follows by an application of the weak maximum principle. $\square$

## Data Availability

No datasets were generated or analysed during the current study.

## Footnotes

1 The exponent range $0<p<1$ in (1.3) corresponds to the fast diffusion case that will be discussed elsewhere (see, e.g., [35]).
